# Experiences of doctoral students enrolled in a research fellowship program to support doctoral training in Africa (2014 to 2018): The Consortium for Advanced Research Training in Africa odyssey

**DOI:** 10.1371/journal.pone.0252863

**Published:** 2021-06-10

**Authors:** Folusho Mubowale Balogun, Yolanda Malele-Kolisa, Sara Jewett Nieuwoudt, Hellen Jepngetich, Jepchirchir Kiplagat, Oyewale Mayowa Morakinyo, Jeanette Dawa, Nomathemba Chandiwana, Admire Chikandiwa, Oluwaseun Akinyemi, Bolutife Ayokunnu Olusanya, Esther Kikelomo Afolabi, Nkosiyazi Dube, Taiwo Obembe, Esther Karumi, Celestin Ndikumana, Justine Nnakate Bukenya, Maria Chikalipo, Sunday Joseph Ayamolowo, Emmanuel Shema, Lester Kapanda, Fred Maniragaba, Felix Khuluza, Henry Zakumumpa, Kikelomo Mbada, Hillary Sang, Emmanuel Kaindoa

**Affiliations:** 1 Institute of Child Health, College of Medicine, University of Ibadan, Ibadan, Nigeria; 2 School of Oral Health Sciences, University of Witwatersrand, Johannesburg, South Africa; 3 School of Public Health, University of Witwatersrand, Johannesburg, South Africa; 4 School of Public Health, Moi University, Eldoret, Kenya; 5 College of Health Sciences, Moi University, Eldoret, Kenya; 6 Department of Environmental Health Sciences, University of Ibadan, Ibadan, Nigeria; 7 College of Health Sciences, University of Nairobi, Nairobi, Kenya; 8 Ezintsha, University of Witwatersrand, Johannesburg, South Africa; 9 Wits Reproductive Health and HIV Institute, University of Witwatersrand, Johannesburg, South Africa; 10 Department of Health Policy and Management, University of Ibadan, Ibadan, Nigeria; 11 Department of Ophthalmology, College of Medicine, University of Ibadan, Ibadan, Nigeria; 12 Department of Nursing Science, Obafemi Awolowo University, Ile-Ife, Nigeria; 13 School of Human and Community Development, University of Witwatersrand, Johannesburg, South Africa; 14 School of Pharmacy, University of Nairobi, Nairobi, Kenya; 15 Department of Governance and Public Administration, School of Governance, University of Rwanda, Kigali, Rwanda; 16 School of Public Health, Makerere University, Kampala, Uganda; 17 Kamuzu College of Nursing, University of Malawi, Blantyre, Malawi; 18 College of Arts and Social Sciences, University of Rwanda, Kigali, Rwanda; 19 College of Medicine, University of Malawi, Blantyre, Malawi; 20 School of Statistics and Planning, Makerere University, Kampala, Uganda; 21 Pharmacy Department, College of Medicine, University of Malawi, Blantyre, Malawi; 22 Department of Political Science, Obafemi Awolowo University, Ile-Ife, Nigeria; 23 School of Arts and Social Sciences, Moi University, Eldoret, Kenya; 24 Environmental Health and Ecological Science Department, Ifakara Health Institute, Ifakara, Tanzania; University of Cape Town, SOUTH AFRICA

## Abstract

**Background:**

The Consortium for Advanced Research Training in Africa (CARTA) aims to transform higher education in Africa. One of its main thrusts is supporting promising university faculty (fellows) to obtain high quality doctoral training. CARTA offers fellows robust support which includes funding of their attendance at Joint Advanced Seminars (JASes) throughout the doctoral training period. An evaluation is critical in improving program outcomes. In this study; we, CARTA fellows who attended the fourth JAS in 2018, appraised the CARTA program from our perspective, specifically focusing on the organization of the program and its influence on the fellows’ individual and institutional development.

**Methods:**

Exploratory Qualitative Study Design was used and data was obtained from three focus group discussions among the fellows in March 2018. The data were analyzed using thematic approach within the framework of good practice elements in doctoral training–Formal Research Training, Activities Driven by Doctoral Candidates, Career Development as well as Concepts and Structures.

**Results:**

In all, 21 fellows from six African countries participated and all had been in the CARTA program for at least three years. The fellowship has increased fellows research skills and expanded our research capacities. This tremendously improved the quality of our doctoral research and it was also evident in our research outputs, including the number of peer-reviewed publications. The CARTA experience inculcated a multidisciplinary approach to our research and enabled significant improvement in our organizational, teaching, and leadership skills. All these were achieved through the well-organized structures of CARTA and these have transformed us to change agents who are already taking on research and administrative responsibilities in our various home institutions. Unfortunately, during the long break between the second and the third JAS, there was a gap in communication between CARTA and her fellows, which resulted in some transient loss of focus by a few fellows.

**Conclusion:**

The CARTA model which builds the research capacity of doctoral fellows through robust support, including intermittent strategic Joint Advanced Seminars has had effective and transformative impacts on our doctoral odyssey. However, there is a need to maintain the momentum through continuous communication between CARTA and the fellows all through this journey.

## Introduction

The African continent lags behind other parts of the world in the generation of scientific knowledge [1, 2]. While the continent disproportionately bears the highest burden of poverty-related and neglected infectious diseases globally, health research originating from the region remains very low [2, 3]. To fill this gap and accelerate sustainable health research capacity in Africa, the Consortium for Advanced Research and Training in Africa (CARTA), was established in 2009 as a partnership involving nine African academic institutions [4, 5]. CARTA focuses on reforming higher education in Africa by helping universities to develop exciting environments that stimulate excellent academic and research pursuits. CARTA also supports promising African scholars at affiliated universities to obtain high-quality doctoral training in public and population health-related fields [6].

The CARTA program, which is multi-disciplinary in nature, is open to university staff in member institutions whose doctoral research questions promote and influence policy change with regards to public and population health issues [5]. Calls for applications run for months and are mostly advertised on the organization’s website and through institutional focal persons who publicize it within their institutions, as well as through other CARTA fellows’ networks. Eligibility for application is contingent upon being less than 40 years in age (for males) or less than 45 years in age (for females) and being a lecturer or research staff with strong passion for capacity building in partner institutions.

The selection process which takes place on a yearly basis starts with interested applicants submitting their applications to their respective focal persons at their institutions. The focal persons then conduct the preliminary screening while the CARTA secretariat notifies successful candidates that scale the hurdle of the first phase of application screening. Thereafter, the candidates are required to complete online tasks and competency-based courses comprising a critical review of a scientific article, numeracy test, critical thinking exercise, plagiarism course, and African Malaria Network Trust (AMANET) basic health research ethics course. These tasks are uniquely developed and revised each year. The final selection of fellows is based on scores by three independent reviewers (including an external reviewer who is not part of CARTA) and is autonomous of the participating institution. The success rate for the application for CARTA fellowship is about 30 percent.

### Organization of the CARTA fellowship program

The organization of the CARTA fellowship program entails completion of various tasks and attendance of Joint Advanced Seminars (JASes) within specified timelines. There are different milestones at different stages of the fellowship which are the prerequisites (known as ‘deliverables’) for the attendance of each seminar [4, 5]. The process commences with the Pre- JAS tasks that CARTA Fellowship nominees are required to complete. These tasks are in the form of assignments and activities within strictly specified timelines. The first month of contact with successful CARTA fellows occurs in JAS-1, which focuses on multidisciplinary research, public and population health and the CARTA approach to capacity development in Africa. Thereafter, an intensive virtual training which focuses on the literature review commences shortly after JAS-1 and ends before the start of JAS-2. The virtual platform supports fellows to develop their literature review sections and the successful completion of this task is a requirement for fellows to attend JAS-2. The second JAS is held in the ninth month of the first year of fellowship and it addresses research methods and the finalization of research protocols. A long interval of 25 months exists between JAS-2 and JAS-3 and this period is normally devoted to data collection by fellows. A prerequisite for JAS-3 attendance is that fellows should have collected most, if not all of the data required to achieve their study objectives because this JAS focuses on the analysis, interpretation and write up of doctoral thesis. The JAS-4 is at 32 months into the fellowship, and it focuses on academic leadership, professional development and life as a researcher after the completion of the doctoral studies.

Each fellow is provided with comprehensive research support to ensure the success of their doctoral research. This support is valued at $100,000 per fellow and it covers the cost of attendance of the JASes and a state-of-the-art laptop equipped with relevant software (including a reference manager as well as both qualitative and quantitative data analysis software). The fellowship also funds internship or trainings to provide specific competencies that the fellows may require for their doctoral research. There is also funding for the conduct of doctoral research including procurement of research equipment and data collection. Provision is made for fellows to be able to have protected writing time (at a location away from distractions) for writing of manuscripts or thesis. The fellows can also apply for tutor exchange whereby a lecturer is paid to temporarily take over their routine responsibilities in the home institution so that they can concentrate on their doctoral research. The fellowship also sponsors conference attendance (where research results are presented) and publication of research findings in peer reviewed journals.

As at March 2021, CARTA had enrolled 229 fellows in 10 cohorts with approximately 23 fellows in each cohort. Each cohort comprises of fellows who were enrolled in a particular year and cohort members are expected to participate in the program together except in some instances when a cohort member applies for a leave of absence because their participation in the activities for the year would not be possible. Such fellows will subsequently join the next cohort to continue the fellowship.

### CARTA theory of change

CARTA is transforming doctoral training in Africa by rebuilding and strengthening the capacity of African universities to produce world-class researchers, research leaders and scholars using the theory of change. CARTA has three strategic priorities namely: building a critical mass of highly trained African scholars at the PhD level, institutionalizing CARTA innovations at key partner institutions and securing the future of CARTA graduates by mentoring them to become leaders in their research fields [4]. It is anticipated that, over time, this will ensure a sustainable and supportive research environment in African universities. It is also envisaged that CARTA fellows will be the agents of change within their institutions [7] as they are expected to institutionalize what they might have learnt from the CARTA PhD curriculum. In addition, CARTA encourages collaborations among fellows and offers postdoctoral re-entry grants to support fellows after their PhDs. Currently, there are 10 cohorts of CARTA fellows among whom are CARTA graduates (those who have completed their PhD) and those who are yet to complete their training.

To the best of our knowledge, only one study has evaluated the CARTA program from the perspective of the fellows and the authors gave suggestions on how the program can be improved upon [6]. One of the recommendations was that fellows should have writing retreats, specifically for doctoral work in order to keep them focused on their program. Some new strategies have been introduced in the CARTA program since this evaluation took place in 2014. This has necessitated a need to re-evaluate the current CARTA program to provide further guidance to improve the program. The first author, FMB, initiated this current evaluation and its aim was to appraise the CARTA program from the fellows’ perspective, specifically focusing on the organization of the program and its influence on the fellows’ doctoral training, as well as individual and institutional development. This is both a formative and summative evaluation because it took place when the fellowship was still on-going for the fellows while some CARTA endpoints had already been achieved by fellows. The overall aim of the evaluation therefore was to help in improving the CARTA program and provide some insights into the effectiveness of the program.

### Good practice elements in doctoral training framework

For this evaluation, the concept of ‘Good practice elements in doctoral training’ that was designed by the League of European Research Universities (LERU) [8, 9] was adapted. Even though CARTA is not a university that runs doctoral programs, her vision for supporting doctoral students is akin to that of universities where doctoral programs are domiciled. Indisputably, doctoral graduates are making significant contributions to innovations and the ability to do so is hinged on a broad skill set. Research organizations who aim to impart this skill set are encouraged to provide structured innovative doctoral training programs that apply the Principles for Innovative Doctoral training [10]. This comprises seven domains (research excellence, attractive institutional environment, interdisciplinary research options, exposure to industry and other relevant employment sectors, international networking, transferable skills training and quality assurance) which seek to make positive impact on the individual doctoral candidate, the educational environment and the global stage. There are four categories of the elements of good practice and they are: Formal Research Training, Activities Driven by Doctoral Candidates, Career Development as well as Concepts and Structures [8]. The use of these elements in the setting up and running of structured innovative doctoral programs is intended “to develop creative, critical and autonomous intellectual risk takers, pushing the boundaries of frontier research”. It is now good practice to organize formal workshop-style development sessions aimed at developing useful research skills as done in CARTA fellowship. In addition, it has become pertinent that doctoral training programs demonstrate effectiveness in developing independence among doctoral candidates by training them to drive initiatives. Also, promoting awareness on career opportunities open to doctoral graduates should be incorporated in doctoral training programs. Furthermore, doctoral training programs are expected to make provision for international and interdisciplinary training structures which can foster useful collaborations.

## Methods

### Study design

An exploratory qualitative study design was adopted. Data were collected in March 2018 through Focus Group Discussions (FGDs).

### Study setting

The study was conducted in Kampala, Uganda during the last of the four residential JASes which was hosted by the School of Public Health of Makerere University. The participating fellows were faculties from the nine partner institutions in Africa that make up the consortium. These are: Ifakara Health Institute in Tanzania, Makerere University in Uganda, Moi University in Kenya, Obafemi Awolowo University in Nigeria and University of Ibadan also in Nigeria. Others are the University of Malawi in Malawi, University of Nairobi in Kenya, University of Rwanda in Rwanda and University of the Witwatersrand in South Africa.

### Sampling technique and sample size

Study participation was open to all attendees of the fourth JAS who could provide the expected information. The eligibility of the participants depended on willingness to participate in the interviews, ability to provide informed consent and attendance of all the four JASes. In all, 21 CARTA fellows participated in the study (10 males and 11 females) (out of the 27 eligible fellows) and they varied in age, area of specialty, home institution and the institutions where their doctoral programs were domiciled. This enabled us to have diverse and robust data. Three FGDs were conducted which comprised an all-male group (five participants), an all-female group (seven participants) and a mixed group (five males and four females). The remaining six fellows did not participate in the interviews for various reasons: four missed the interview time, one was unwell and the last fellow had to attend to urgent domestic issues.

### Study instrument

An interview guide was developed for data collection. Face validity of the instrument was carried out by faculties who were not CARTA fellows in one of the partner institutions while content validity was ascertained by two CARTA staff. The guide had one broad question “Can you tell us what your experiences have been with the CARTA fellowship program?” Fellows who participated in the FGD were asked to fill a self-administered questionnaire detailing their doctoral background and research achievements.

### Data collection

Two experienced independent female research assistants were trained on the use of the developed interviewer guide. The research assistants received training to familiarize themselves with the study, its background, aim and their roles and responsibilities in the process of data collection. One was the facilitator of the FGDs while the other was the timekeeper and note taker. The discussions were conducted in a designated quiet room and all were conducted in English. Each participant gave verbal informed consent and discussions were digitally recorded. To ensure anonymity, participants were identified only by codes. After each discussion, a summary of key issues was communicated to the participants for them to confirm their responses were correctly captured as part of member checking.

### Trustworthiness of the data

Using the four criteria for ensuring trustworthiness of qualitative data [11, 12], the following steps were taken:

#### Credibility

The research instrument was developed by the CARTA fellows and staff while the research assistants were taught about the workings of the CARTA program during their training. This was to ensure that all were familiar with the study group and to provide a good understanding of the data. Almost all the CARTA fellows participated in the study, thus providing a very diverse data source and generating robust data. Probes were used during data collection process to ensure detailed data was obtained and member checking (by paraphrasing what the fellows said at intervals and summarizing the content of each session for them to decide if their responses were correctly captured) after each focus group discussion helped to ensure the correctness of the data.

#### Transferability

Details about the study participants, study design, data collection procedure and analysis have been provided to aid the understanding of the results and help in the comparison of the findings with similar studies.

#### Dependability

The research process has been described in detail which should be helpful in reproducing similar research.

#### Confirmability

All the study participants were doctoral students at an advanced stage of their studies and all had gone through the four JASs, this made them a homogenous group regarding their experiences with CARTA. This informed the use of FGDs in the data collection for this study.

### Data management and analysis

The recorded information, field notes and transcripts were kept in a lockable cupboard accessible by the researchers only. The computer with the data had a password known by one of the researchers (FMB). Soon after data collection, the research assistants transcribed the recorded data verbatim. A mix of deductive and inductive coding was applied and the key elements of Good practice in doctoral training framework were used to identify deductive themes. All fellows assigned to data analysis read the full transcripts to get a sense of the data and important issues. Fellows were then assigned specific themes to explore with inductive coding, to draw out nuances. These were cross-checked by a second member of the analysis team until agreement was reached for the final framework. This was used to code the remaining transcripts while paying attention to emerging issues from the data. To ensure accuracy, each transcript was reviewed by comparing it with the tape-recorded information. The codes were organized into various categories based on their similarities and differences which were then brought together as overarching themes grouped under each element of good practice in doctoral training framework. The doctoral characteristics and research achievements of the fellows were summarized using proportions and medians.

### Ethical considerations

This study was exempted from ethical approval by the Oyo State Research Ethics Review Committee in Nigeria because it is a project evaluation research. The fellows understood the procedures of the study and were free to exit at any stage of the research without facing reprimands. All the fellows were adults who participated voluntarily in the research after giving their informed consent. Permission was obtained from them for the discussions to be recorded. Confidentiality and privacy of the participants were ensured by conducting the discussions in a private place and using only codes for identification of the participants.

## Results

Of the 21 fellows who participated in the FGDs, 19 filled in the self-administered questionnaires (9 males, 10 females). These 19 fellows were drawn from six African countries (Kenya, Malawi, Nigeria, Rwanda, South Africa and Uganda). Five fellows (26.0%) had undertaken PhD studies in African countries other than their countries of residence. Most of the fellows (n = 14), had been in the CARTA program for 3 years, four had been in the program for 4 years and 1 fellow had been in the program for 5 years (Table 1).

**Table 1:** Characteristics of CARTA fellows who participated in the study.

Item	Description	Number (percentage)
1. Gender	Male	9 (47%)
	Female	10 (53%)
2. Country of residence	Nigeria	7 (37%)
	Kenya	4 (21%)
	South Africa	3 (16%)
	Malawi	2 (11%)
	Rwanda	2 (11%)
	Uganda	1 (5%)
3. Undertook PhD outside country of residence	Yes	5 (26%)
	No	14 (74%)
4. Years within CARTA program	3 years	14 (74%)
	4 years	4 (21%)
	5 years	1 (5%)

The experiences of the JAS 4 participants with the CARTA fellowship program were diverse across the three groups and it was influenced by the practical skills gained from the fellowship and the overall appreciation. The major themes that emerged were in relation to the impact on individual knowledge and skills, their doctoral training and the overall views of the structure as well as the operation of the program. Using the concept of Good practice elements in doctoral training, the major themes and subthemes that emerged were grouped into the four categories of the concept as follows:

Formal Research Training

- Appraisal of the Joint Advanced Seminars (JASes)
- Impact on individual knowledge, skills and self confidence
- Impact on doctoral program

Activities driven by doctoral candidates

- Improvement in research outputs

Career Development

- Change in institutional practices
- Improvement in teaching skills
- Improved organizational skills
- Future ambition

Concepts and Structures

- Exposure to multidisciplinary research
- Specific CARTA support

### Formal research training

#### Appraisal of the Joint Advanced Seminars (JASes)

Participants shared their experiences of the four JASes they attended and these experiences varied from one individual to another. While some were focused on the specific conditions of the seminars, many were related to the organization of seminars, expertise, and communication during and in-between seminars. Overall, fellows viewed the JASes as the nidus and the springboard which birthed the transformations that they experienced because both research and teaching skills were gained from the JASes. This was aptly described by one of the fellows in this quote.

*“I will personally describe CARTA journey as an onion*, *when you have an onion and you keep on peeling it layer by layer as it gets inside you get inside you have fresher leaves because each JAS I attend*, *I keep on hearing about new opportunities*. *JAS 1 and 2*, *I got to know so much about the internships training*, *JAS 2 they were also telling me about writing grants*, *because those are opportunities*, *it is a well-designed program…even if I go to do my PhD in outside countries*, *I cannot have a better package because I have access to almost 100% of the facilitators”*

(P6, Female FGD)

The fellows compared the four JASes and JAS 3 was rated as the best. Some fellows felt JAS 1 was confusing while JAS 1, 2 and 4 were seen as too packed and stressful. However, the stress of JAS 1 was seen as a teething problem for fellows as described in this quote:

*“I think some of the confusion that was in JAS 1 is that most of the confusion is healthy…I think it’s appropriate*. *If you came into CARTA*, *you need to change things in the first 6 months that you start.”*

(P2, Mixed FGD)

JAS 3 was seen as the model for balancing teaching sessions with time for individual work and reflections. This view is summarized in the quote below.

*“I think it is one of the JASes (JAS 3) where you can see the output*, *you go in with inputs and after coming out you either come out with the drafts*: *a draft manuscript*, *a draft of analysis… not so many lectures… few assignments and if it was assignment it was purely related to your own work something that makes you continue working on your own work*.

(P1, Female FGD)

Fellows across the FGDs felt strongly that the other three JASes could be improved by scheduling more time for personal reflection and work. Embedding leadership classes across all JASes, including self-reflection, was also recommended as a best practice rather than emphasizing it in JAS4. The quality of the facilitators was also rated high by the fellows. The diverse institutions they came from and the various skills they brought on board were seen by the fellows as one of the strengths of the CARTA program. The ease of access to the facilitators was also stressed in the quote below.

*“If I can add something about the facilitators we come from institutions where professors are there you can’t reach them/not accessible*, *CARTA has brought all her facilitators to our level*, *you can call each other by first name*, *everybody will understand you every one will listen to you*, *you can work together*, *you can eat together on the table.”*

(P1, Female FGD)

#### Areas which need improvement

Many participants felt that there were different challenges which should be addressed to improve JASes related experience. The low points they alluded to were related to planning, organization and logistical problems of the JASes and they offered useful suggestions to improve on them.

*Planning*. Timing for JAS 2 and the expected deliverables need to be synchronized as exemplified in the quote below.

“*Some people actually got their ethical clearance before JAS 2 and that’s another thing that should need to be clear because some of us had ethical approval before coming for JAS 2 and in JAS2 they were like trying to teach some things which wasn’t going to be possible”*.

(P4 Female, FGD)

Participants expressed concern that there was a long break between JAS 2 and 3. Participants said that during this period of time fellows barely received any communication from CARTA secretariat. The deliverables for JAS 3 admittance were re-echoed a few months to the seminar but this would have been helpful if it was sent much earlier.

“*I think the gap between JAS two and three is a lot and actually the explanation they give is that they expect us to be in the field but at times it’s like we get lost… some deliverables were not clear cut”*.

(P7 Female, FGD)

*Communication*. Clear and regular communication between the different seminars was another key theme. Clear lines of communication from the CARTA Secretariat were desired, especially when there were changes in personnel. Communication to monitor progress and motivate fellows between the seminars was again recommended to enhance fellows’ experiences. Fellows suggested that during the 18-months gap between JAS 2 and 3, sending out JAS3 requirements and timelines earlier would be helpful to fellows. Besides, thorough assessments of fellows before JAS3 admittance, and support for data management (especially for specialized types of data) during this period were also suggested to better leverage gaps.

#### Impact on individual knowledge, skills and self confidence

It emerged from across all three focus group discussions that fellows gained practical and tangible skills in academic writing and in preparing grant proposals, as it was put by one of them:

*“Apart from the core skills*, *there are also the transferable skills that I have got through this process*, *I think when we are in academic institutions the goal is around getting a PhD whereas in CARTA there are a lot of other key transferable skills like grant writing*, *networking and things that seem not important but actually do help a lot”*.

(P4, Mixed FGD)

In this same viewpoint, the fellows also mentioned the research skills that were learnt through a series of JASes and that impacted on their research activities with improvement in their research design and the use of both quantitative and qualitative data analysis software. To this end, one fellow stated that:

“*There were practical skills*… *how to use PubMed efficiently*, *how to do searches*, *data management using STATA and NVIVO*. *There were practical things around that and how to refine a research question”*.

(P2, Mixed FGD)

In the same way, the impact on the knowledge and skills gained in academic writing was highlighted in the fellows’ ability to write sound literature review, as it was mentioned by one fellow that:

*“I can say when I began the fellowship*, *I didn’t have good research skills but will say that I have turned around*. *With research*, *knowing how to write a proposal*, *literarily knowing how to do literature review not just review but critical review of literature….”*

(P1, Female FGD).

The fellows talked about the change in their views about research and the ability to handle research activities. The CARTA fellowship was considered to have made them to be conscientious and confident as some of them said that:

“…*I am more confident*, *more decisive…they (CARTA) have really supported us”*

(P8, Mixed FGD).

This was reiterated by a fellow from the mixed group who revealed that:

“*I think I am more confident now to join the research world and as I’ve said earlier*, *I realized that you don’t do research by sitting in the corner of your room”*

(P4, Mixed FGD).

#### Impact on doctoral program

Overall, CARTA fellows expressed their contentment towards the skills and knowledge gained and spoke regarding the influence of these on their experiences during their doctoral training.

The participants appreciated the positive competition that CARTA has instilled among its fellows. They felt that the progress made by other fellows was an inspiration to them and would try to attain similar achievements. This was illustrated in this quote:

“…*it induces a sense of benchmarking so*, *if my other colleagues have moved a certain stage*, *I also need to run and it’s only here in such a situation that actually lies the capacity*, *at times you think*, *I cannot do this thing*, *but if your other colleagues are doing it*, *if they are winning grants*, *you can also win grants*, *if they are also publishing*, *you can also publish”*.

(P8, Mixed FGD)

### Activities driven by doctoral candidates

#### Improvement in research output

Prior to joining the CARTA program, five (26%) of the 19 fellows who filled the questionnaire had not made oral presentations at scientific conferences and 12 (63%) had not made poster presentations at scientific conferences. Also, seven (37%) had not published peer reviewed articles in scientific journals, 10 (53%) had not undertaken public engagement activities to share their research findings with the general public, and 14 (74%) had not won research grants (Table 2). By completion of the CARTA fellowship there were improvements in the frequency of scientific presentations, publications and acquisition of research grants as shown in Table 2.

**Table 2:** Research achievement of CARTA fellows from commencement of the fellowship till March 2018.

Item	Number (percentage)	
	Before joining CARTA	As at March 2018
Had not made oral presentations at scientific conferences	5 (26%)	1 (5%)
Had not made poster presentations at scientific conferences	12 (63%)	2 (11%)
Had not published a peer reviewed article in a reputable journal	7 (37%)	1 (5%)
Had not undertaken public engagement activities	10 (53%)	3 (16%)
Had not won research grants	14 (74%)	3 (16%)

The present evaluation is also an example of an activity driven by the doctoral candidates as its conceptualization and execution were stirred up by the sharped research skills obtained from the CARTA fellowship.

During the CARTA fellowship, individual fellows had a median of three (range of two to five) oral presentations and one (range of one to three) poster presentations at scientific conferences, published a median of four (range of two to eight) peer reviewed journals, engaged the lay public on their research findings twice, and had won a median of one (range of one to two) research grants as co-investigators or key personnel (Table 3)

**Table 3:** Performance of CARTA fellows from commencement of the fellowship till March 2018.

Item	Median (interquartile range)	
	Before CARTA fellowship	During CARTA fellowship
Number of oral presentations at scientific conferences	1(1–3)	3(2–5)
Number of poster presentations at scientific conferences	0(0–1)	1(1–3)
Number of peer reviewed article in a reputable journal	2(0–4)	4(2–8)
Number of public engagement activities	0(0–1)	2(1–3)
Number of successful research grant applications	0(0–0)	1(1–2)

### Career development

#### Change in institutional practices

Many fellows believed they had become change agents within their own institutions and this has resulted in shifting of institutional practices. This is exemplified by the change a fellow introduced in the teaching about plagiarism as shown below.

“*When I went back to the institution*, *I was very curious about the plagiarism aspect so every time I would send a proposal*, *I would request a soft copy*, *then go through it*. *I run through it in Turnitin and I found everything is plagiarized*, *so I became a bit critical*. *And I at some point felt like this is not fair to students and … what I am doing is not fair*. *So I requested to be part of the team that teaches on ethics and plagiarism”*.

(P3, Female FGD)

This argument was not different from a participant from the mixed focus group who started teaching about research skills that were not being taught in his institution as shown in this quote:

“*At my own University I have been able to be a resource to other PhDs and faculty who are interested in putting together proposals to come together and talk through the process …my university has taken this seriously*, *and I have had to pass it on as part of the things that CARTA has encouraged us*, *to teach forward or apply what we were learning*…*I now teach NVIVO at my University”*.

(P6, Mixed FGD)

#### Improvement in teaching skills

Participants appreciated that the skills gained have supported their PhD research process as well as their teaching responsibilities in their institutions. The fellows mentioned that they acquired teaching skills and approaches from the CARTA model that has influenced their own teaching at their home universities and research institutions. One of the fellows’ views read:

“*One aspect I would like to emphasize is the teaching methodology*. *So as teachers*, *you know most of us are lecturers at the university*, *so I have learnt the teaching methodologies and approaches from different facilitators as we have made across this journey from different JASes”*.

(P9, Mixed FGD)

There has also been improvement in the content of what the fellows taught in their various institutions as a result of the skills that they learnt from the CARTA JASes as seen in this quote.

*“…I had never done qualitative research in my life*, *and in my PHD I am doing mixed study research*. *And even in my university I have been invited on different fora to come and teach qualitative research*. *So*, *I was like wow; how many years…and Iam now teaching people?”*

*(P6*, *Female FGD)*

#### Improved organizational skills

In addition to this, the CARTA methodology during the JASs also taught fellows organizational skills because they had to plan their time to cope with tight schedules and timelines as it was mentioned that

“*CARTA has positively impacted my life*, *I have learnt and now have skills on how to organize myself*, *meet deadlines*, *I have been able to work in a competitive environment with some other people to achieve set goals*, *I think that is a plus for me in CARTA”*.

(P7, Mixed FGD)

#### Future ambition

Fellows shared their ambition for the next five to 10 years. The aspirations of participants include wanting to be a research leader, grants winner, a great mentor, prolific author, capacity builder and professor. Many of the fellows hope to influence younger colleagues as well as government policies both locally and internationally. According to a participant,

“*I see myself becoming a professor having big research grants*, *… having many mentees that actually make an impact in the field”*.

(P1 Female, FGD)

In the words of another respondent,

“*I see myself becoming a don…”*

(P3, Mixed FGD).

In addition, some respondents expressed their desire to become change agents, carry out policy-influencing research and make their contribution to societal development.

“*I also see myself as actually being a research leader; doing research and influencing policy… being a change agent in society*…”

(P3, Mixed FGD)

“…*Having research findings that can really influence policies not just in my country but Africa and even outside Africa*…”

(P1, Female FGD)

Furthermore, some CARTA fellows would like to take up university administrative responsibilities and be involved in capacity-building. A respondent expressed the desire to be:

“… *a change agent*, *facilitating the next generation of researchers and top-class researchers and scientists from my institution…CARTA has been providing platform to achieve that.”*

(P3, Mixed FGD).

Also, one of the respondents stated that

“…*I’m able to commit as a leader*, *a person that will be assisting the others a lot to get their career paths”*.

(P3, Mixed FGD).

Another respondent added:

“*After going through the CARTA experience*, *I hope I will be able to build capacity.”*

(P1, Female FGD).

Most discussants believed that they have learnt a lot from the CARTA fellowship which would put them in a good footing to achieve their future ambitions:

“…*I have learnt from CARTA what I would like to do in my institution that wasn’t there.”*

(P1, Female FGD)

### Concepts and structures

#### Exposure to multidisciplinary research

Aside from the exposure to the teachings and examples from the CARTA set up, the fellows were able to interact with colleagues from different cultures and countries resulting in collaborations in different areas and contributing to their transformation. It was obvious from the discussions that some of them did not understand the nature of multidisciplinary research initially as one of them stated that:

“*For me CARTA has been quite an experience*, *it has changed the way I think*, *the way I look at things*. *Okay before I joined CARTA I used to think of research in a straight way just in my field*, *I didn’t think of it beyond—but this multidisciplinary approach brings people together; you now see…how a mathematician fits into my thinking into my line of research; this is how a social scientist fits in*, *this is how*, *so it has expanded my thinking of research”*.

(P2, Female FGD)

In addition, the CARTA program has offered the fellows a platform to work in multidisciplinary teams which has helped them to hone their skills in team work and appreciating the beauty of multidisciplinary collaboration. To this end, it was stated that:

*“…the range of disciplines that we have had to interact with in our cohorts have actually broadened my horizon*, *with respect to understanding how to relate with other people and how different disciplines can enrich research when you have different disciplines being involved in research project*. *That has actually helped me to appreciate that quite well”*.

(P**2,** Mixed FGD).

The CARTA multidisciplinary approach towards research practices was seen as a springboard to fostering multidisciplinary collaboration within and between African institutions. To this also the fellows acknowledge that it was an opportunity to learn from different people:

“*…CARTA has been a catalyst for change among intellectuals*. *You know intellectuals coming from teaching in the universities*, *we were in our comfort zones and so actually CARTA pulled us out from that comfort zone putting you somewhere where you are made to learn from your fellow friends having different backgrounds and that actually to me meant quite a lot*, *but again my experience has been that CARTA encourages multidisciplinary kind of PhD programing and that actually is good because I don’t think I have read any other program initiated in Africa that actually encourages multidisciplinary approach in term of doing research”*

(P5, Male FGD)

This fellow also added:

“…*having been exposed to this kind of training where you have met different people with different experiences*, *your own fellows*, *from different universities with different experiences*, *that actually made me look at myself as someone who can always learn from others because you know research is not a one-off thing”*.

(P5, Male FGD)

Another fellow put this multidisciplinary concept in a different light and stated that:

“*…the fact that we have been in various countries and gotten the exposure to different contexts like the surveillance sites*, *different institutions … has given me a lot more respect and understanding of how capacity building is in the continent*, *what the challenges really are and where we are coming from has made me much more inspired has made me much more efficient and has made me much more committed to the visions of CARTA and not just the individual …so that shifted the kind of thinking that I had”*.

(P6, Mixed FGD).

The multidisciplinary concept has given the CARTA fellows networking opportunities which they see as a long-term platform that would outlive their CARTA fellowship experience. This was aptly stated by one of the fellows below.

*[CARTA] has taken me out of my country it has moved me a bit around Africa so it has even widened and broadened my view and exposure to experience*. *In terms of African networks*… *I see myself collaborating with most of my peers even on CARTA program and even beyond CARTA when I am done with my PhD*.

(P2, Male FGD)

#### Specific CARTA support

The fellows talked about specific supports of the CARTA program which impacted on their doctoral research and career. Specifically, the provision of funds for research helped the fellows to conduct good research for their doctoral studies as shown in the quote below.

*“But with CARTA*, *I was able to access the research funds and I was able to acquire those equipment to carry out my research and I think the finance from CARTA has been quite helpful.”*

(P4, Male FGD)

The internship as well as funded protected writing time away from work and home to focus on writing of PhD thesis and manuscripts were mentioned as highlights as seen in these quotes.

*“You know when I got the protected writing time*, *I was able to utilize my time wisely*. *I designed my own writing retreats where I was able to go for a week or two where I would pay for myself to be away to just write and push my PhD work.”*

(P1, Male FGD)

“*Okay after JAS3*, *I got an internship from CARTA to go for a training*, *so I had 6 weeks dedicated and it was very helpful*, *I also had access to my supervisors.”*

(P2, Mixed FGD)

The required deliverables for the different stages of the fellowship were said to be useful tools that have kept the fellows on track with their PhD research. This was captured aptly by one fellow below.

*“Well as to the deliverables… the fact that we had goals to attain has been very helpful*, *I am worried that now we are in JAS4 for those of us who haven’t finished and what will be the push…”*

(P4, Mixed FGD)

Some female participants also appreciated the fact that they were able to bring their infants to attend the JASes with the full support of CARTA. One of them stated that:

*“Yah I just want to talk about the policy to be able to bring up my child to JAS1… so*, *I appreciate it because that’s the only way I could have done the fellowship.”*

(P6, Mixed FGD)

The other supports that were mentioned in all the FGD groups were the cohort system, monthly stipends, conference and publication funding.

## Discussion

The CARTA odyssey have obviously has left indelible impacts on us in different areas aside from the influence it had on our doctoral training. This has made us to have a resolve to transform not only our immediate institutions and environment, but also to become players in research capacity building in the global scene.

The intensive JASes was a major contributor to the building of our research capacities as CARTA fellows. These JASes were designed strategically to provide specific support and direction for the fellows at different milestones in the PhD journey [5, 6] and they were conducted in conducive environments, away from the home institutions. This helped fellows to focus without distractions. The traditional apprenticeship model that is seen in many African institutions for the doctoral program does not provide adequate disciplinary training required for doctoral studies and this can reduce the quality of PhD output on the continent [13]. Introduction of course work which aims to provide research skills that are required for baseline knowledge is necessary to fill this gap. This will also be a melting pot for the multidisciplinary background of doctoral candidates and help build a solid foundation for multidisciplinary collaboration in future research. Fisher et al. reported that doctoral students in sub-Saharan African universities who participated in a writing course were more likely to have more research publications than those who did not [14] and this underscores the importance of formal research teaching in doctoral studies. This therefore implies that other organizations providing support for doctoral programs in Africa will have greater impact if research training is incorporated in their core activities.

The extra support for nursing mothers which enables them to attend the JASes with their babies and caregivers should be applauded. It is a good way of responding to the gender inequality seen in African academia [15, 16]. African women whose institutions have policies that support women tend to complete their PhD faster than those who do not [14]. This support is necessary because of the complex gender specific factors that make the pursuit of a PhD difficult and unattractive for the female gender. The creation of a conducive environment for female fellows to continue the care of their infants during JASes encourages active participation in the seminars and gives such mother-fellows the opportunity to continue their doctoral studies without disruptions. This is an apt response to the need to tailor doctoral studies to meet Africa’s social peculiarity. It would help doctoral programs to be relevant and impactful on the continent.

The first JAS was built on basic research competencies and the other JASes continued the development of the capacity building by offering the relevant skills which were streamlined to provide the mastery required at each step of the doctoral training [5]. The other programs such as the internship to learn specific skills, protected writing time, and the online writing training also added extra color to the doctoral training process. These greatly improved the quality of the training that fellows received and also prepared them to be research leaders who can stand shoulder to shoulder with colleagues across the world–thus, achieving CARTA’s aim of building world class researchers in Africa [4].

The improvement in our teaching skills was an indirect benefit of the research training that we had. During the JASes, the facilitators used different pedagogical skills with resultant experiential and participatory learning in contrast to the didactic lectures which characterize most institutional learning in Africa [5]. Resources for pedagogical development are low in African universities and many university teachers do not have any formal pedagogic training prior to their employment [9]. This clearly reduces both the quality of education that African universities have to offer and the job satisfaction of the teachers. The teaching skills in the JASes were a hidden curriculum, but it was easy for us to learn these teaching skills because almost all of us were university teachers. This way, we were able to develop our teaching skills simultaneously. This has consequently affected the way we teach in our various home institutions and has facilitated the transference of the skills we were taught. This transfer of skills was not limited to students but other faculties in our home institutions benefitted as well. Furthermore, we are already taking up responsibilities to ensure that the standard of practice in public health research is improved in our institutions as exemplified in the account by the fellow who took it upon herself to teach students about plagiarism and the one who decided to train others on how to use the Nvivo software. These are indications that CARTA’s investment on fellows is already having a ripple effect as it contributes to research capacity building in the various partner institutions [2]. This is in contrast to the costly traditional training abroad which has led to brain drain [3, 17] or the system in which the students received training abroad which are irrelevant to their home countries [18, 19]. There is also the practice of placement of foreign experts in African universities for limited period of time [3] but the effectiveness of this system is not really clear.

In addition to the transferable research and teaching skills that we learnt from CARTA, we have been launched into the world of research. The skills we learnt were necessary ingredients for the development of good research proposals and this has the propensity for attracting research grants. Funding for research in Africa is currently abysmally low [18], but with good grant writing skills, the probability of getting good funding for research is increased as evident in our experience. We were also taught leadership skills which can be put into use in developing productive and cohesive research teams. The exposure to other disciplines through our interactions with other fellows from diverse backgrounds within and between CARTA cohorts has made us to appreciate other disciplines and to understand their contribution to research [5]. This reflected in the quality of doctoral research that we carried out as we were equipped to use diverse research skills such as the skills for mixed study designs which were new to most of us. This also made the focus of our doctoral researches to fit into our local contexts and they were highly innovative, making them more relevant in providing solutions to African public health problems [2, 18]. The understanding of social determinants of disease which was introduced in JAS 1 is a useful tool that could be of tremendous help as we work in multidisciplinary research teams.

The multidisciplinary value that CARTA promotes is a necessary requirement which can provide practical solutions to the public health problems plaguing Africa [5]. This is because these problems have multiple contributing factors just as highlighted in the social determinants of health [18]. Effective interventions that can address these problems require a multidisciplinary approach which will make the problem to be seen from different perspectives and yield holistic solution-approaches. The health problems in Africa require such home-grown all-encompassing approaches for the appalling health indices of the continent to be improved [2]. This also holds the key to economic development, which has evaded the continent for decades [17].

The cohort system used in CARTA also made the interaction across disciplines to be possible, easy and motivational. This is seen in the encouragement that comes with the sharing of experiences and resources among cohort members. Schriver and colleagues who studied the effect of twinning doctoral students of the University of Rwanda and Aarhus University in Denmark reported that twinning of PhD students led to successful completion of the programs with promotion of collaboration [20]. Thus, the cohort system, which promotes peer mentoring [21] provided a platform that is fostering deep interactions among the fellows, thereby giving extra support that has helped to keep fellows on track. It has also enabled the determination and encouragement to pursue quality research as well as timely completion of the program. This present study is an example of the networking skills and collaborative approach to research that the cohort system of CARTA has engendered in us.

CARTA has successfully birthed change agents in research and training in the different partner institutions across Africa as seen in this study. Africa’s contribution to health research and published articles which have been shown to be low [2] indicates low research capacity [1], but the changes that the CARTA experience has afforded us would definitely help us in contributing to the turning of the tide towards improved health research and publication in Africa. The institutional capacity building can be strengthened through institutionalizing the CARTA program in the different partner institutions, an initiative that was commenced recently.

The aim of good practice elements in doctoral training was achieved through the inculcation of these skills based on the structure of CARTA with the resultant impact on the activities of the fellows, including their career. The CARTA model, however, took these a step further by putting into consideration the peculiarity of the African setting in the design of her structures. The major challenges mitigating against doctoral programs (like the distractions in the fellows’ home institutions as a result of huge workloads, the responsibility of women nursing their babies and inadequate access to mentors) were addressed by the CARTA structure aside the traditional provision of research funds and research training that most traditional doctoral fellowships offer. These earlier supports have been ineffective in meeting the needs of Africa. Other programs that support doctoral studies in Africa can emulate the CARTA model for better effectiveness. This will ensure better value for the resources spent on these programs.

One of the major contributors to CARTA’s achievement over the years is the feedback system. Another key contributor is the evaluation of the program by the participants during each of the JASes [5]. This study provides additional participatory evaluation which can further improve the CARTA program. Continuous improvement is needed to keep the program in the optimum state. We recommend quarterly reminders about the deliverables to achieve the required milestones between JAS 2 and 3 as well as to keep fellows on track during the long interval between these JASes. This can help fellows to be more focused and organized bearing in mind that there are other responsibilities competing for attention at the home institutions during this period. These deliverables could also be better synchronized with the doctoral procedures of the partner institutions so that the fellows can maximize the benefits of the CARTA program.

### Limitations of the study

This study has some limitations. Firstly, it was conceptualized by the CARTA fellows and some of its staff and the data were also analyzed by them. This could introduce some bias in the design and the report, but considerable efforts were made to concretize the trustworthiness of this study. This was achieved by using external data collectors who were trained on the workings of CARTA before the data collection process, member checking after collection of each data and ensuring responsible conduct of research at every stage. Secondly, this study was conducted among only one cohort of fellows attending their final JAS. Interviewing more groups of JAS 4 attendees could give more data with a probability of more variations in the findings. These limitations may not allow the generalizability of the study’s findings to other similar doctoral programs.

## Conclusion

In conclusion, the CARTA model has demonstrated how elements of good practice in doctoral can be structured for the building of research capacity through the CARTA doctoral fellowship across African institutions. This has positioned us to become research leaders, mentors and change agents through the opportunity given us to undergo an unconventional doctoral journey. We have been adequately equipped for the academic and research clime as evidenced by our research and career achievements with aspirations and dreams to be forces to reckon with, not just in Africa, but also in the global scene as research leaders, mentors and change agents. Thus, fulfilling CARTA’s aim of producing world class multidisciplinary African researchers who would have positive impact on Africa’s population and public health.

## Supporting information

S1 Transcript Male CARTA fellows’ interview. (DOCX)

S2 Transcript Female CARTA fellows’ interview. (DOCX)

S3 Transcript Mixed group interview. (DOCX)
