# Correction: Experiences of doctoral students enrolled in a research fellowship program to support doctoral training in Africa (2014 to 2018): The Consortium for Advanced Research Training in Africa odyssey

**DOI:** 10.1371/journal.pone.0298875

**Published:** 2024-02-09

**Authors:** Folusho Mubowale Balogun, Yolanda Malele-Kolisa, Sara Jewett Nieuwoudt, Hellen Jepngetich, Jepchirchir Kiplagat, Oyewale Mayowa Morakinyo, Jeanette Dawa, Nomathemba Chandiwana, Admire Chikandiwa, Oluwaseun Akinyemi, Bolutife Ayokunnu Olusanya, Esther Kikelomo Afolabi, Nkosiyazi Dube, Taiwo Obembe, Esther Karumi, Celestin Ndikumana, Justine Nnakate Bukenya, Maria Chikalipo, Sunday Joseph Ayamolowo, Emmanuel Shema, Lester Kapanda, Fred Maniragaba, Felix Khuluza, Henry Zakumumpa, Kikelomo Mbada, Hillary Sang, Emmanuel Kaindoa

There are errors in the Funding section. The correct Funding statement is: This research was supported by the Consortium for Advanced Research Training in Africa (CARTA). CARTA is jointly led by the African Population and Health Research Center and the University of the Witwatersrand and funded by the Carnegie Corporation of New York (Grant No. G-19-57145), Sida (Grant No:54100113), Uppsala Monitoring Center, Norwegian Agency for Development Cooperation (Norad), and by the Wellcome Trust [reference no. 107768/Z/15/Z] and the UK Foreign, Commonwealth & Development Office, with support from the Developing Excellence in Leadership, Training and Science in Africa (DELTAS Africa) program. The statements made and views expressed are solely the responsibility of the Fellow. CARTA had no role in the study design, data collection and analysis, decision to publish, or preparation of the manuscript.
