# Supplementary material for: Experiences of doctoral students enrolled in a research fellowship program to support doctoral training in Africa (2014 to 2018): The Consortium for Advanced Research Training in Africa odyssey
Source: PLoS One. 2021 Jun 10;16(6):e0252863. doi: 10.1371/journal.pone.0252863 (PMC8191976; doi:10.1371/journal.pone.0252863)
Supplement: S1 Transcript — (DOCX) [file pone.0252863.s001.docx]

**CARTA FGD**

**DATE OF INTERVIEW: 12/03/2018**

**CATEGORY OF FGD: MALE FELLOWS**

**MODERATOR: KOMUHENDO ROBINAH**

**NOTE TAKER: NAKAYIMA RESTY**

**DURATION OF INTERVIEW: 1Hour: 19minutes**

**KEY**

**Mod**: moderator

P1, P2, P3… Respondent

**DESCRIPTION OF INTERVIEEW**

**TRANSCRIPT**

**Mod: We shall request you to share with us or describe your experiences with CARTA.**

P2: Okay well if I would start I think the experiences have been if I would say it is very humbling and superb I think it is a project and it is a scheme that has been very, very good and nice the reason I am saying that is because even though I am not yet done with my PhD I can see the end in sight for me it makes it a reality that one can finish my PhD with in four years and I say that also because I had been on PhD program before in my institution which I was on for two years , I know I did not get anything down so when it comes to the speed and time duration to which CARTA program helped me to finish in four year I think in that it is a very good idea it is a beautiful and well put up program , more importantly I think something that has been very good in terms of CARTA, CARTA has been the access to exposure to international people who have been able to impart in our lives it has taken me out of my country it has moved me a bit around Africa so it has even widened and broadened my view and exposure to experience in terms of African being on the hope I see myself collaborating with most of my peers even on CARTA program and even beyond CARTA when I am done with my PhD and then this is not something you get from your institution in your country if you are to do your PhD in your home country. And I must not forget the fact about the financial support goes a long way while financial support even when you feel like doing anything concerning PhD program remembering those milestones remembering things you got to lose remembering what has been invested in you that also in so many ways helps you to keep going even when the morale and the zeal is very low. So for me those are my experiences in a nut shell, it has been beautiful the experience, has been nice.

**Mod: What do the rest have to share on their experiences with CARTA?**

R: This experience is it bad or good experience?

**Mod: It can be both, it can be bad or good.**

P1: I think I would say the frame work before the experience for me I think my experience with CARTA first is that it keeps Africans with in Africa. I am one person who does not like programs that that would take me to Oxford Wales, Harvard where ever. I believe in investing with in the African continent because I keep thinking what is Africa doing they are many programs that are natured from Africa but due to lack of financial support talents in Africa so we are forced to take on those because of lack of financial resources so I think that to me was the first thing that made me apply for CARTA because I remain in Africa , And remain in the continent. Other thing in terms of the experiences so far everything has gone well I know I will be able to complete in less than four years or less than 48 months but am gland to say that with the support of CARTA through various program I have been able to say that the PhD is at the bridge yes if it was not for CARTA I would imagine there would be too much commitments like teaching in the our institution, supervision but CARTA put that load off for us so that students should focus on our PhDs . And the other program that I benefited most was the protected writing time. You know when I got the protected writing time, I was able to utilize my time wisely I designed my on writing retreats where I was able to go for a week or two where I would pay for myself then to be away to just write and push my PhD work , I think on the negative the thing I would say I think there is less advocacy on CARTA focal persons in our institutions I think it is high time that we don’t have just focal persons we need a focal person who is takes CARTA fellows issues seriously and that person should be a person who goes to the institution heads on like this is a CARTA fellow these are your deliverables and these are your roles in this institution these is not a departmental program as far as I know it is kind of a university or centered program that is pushing different objectives so I so I think the focal person are those are the ones so far I feel they are not doing so much especially for the CARTA fellows I think we need to step up on those and may be they are no incentives that they are just doing for the sake of doing it , I think there is should be something that CARTA does that at least we see a change on those.

**Mod: Gentlemen your experiences.**

P3: I think they have exhausted the experiences but what we have for CARTA are the stipends as always they have spearheaded and as there are always milestone within, and with the mile stones they will always push you toward achieving your goal so with those milestones we have been able to achieve some of the goals not all of them but they were pushing us to achieve the goals in between so in a sense I think we would just be repeating what the other two have spoken.

Mod: **Yes the experiences may be similar but we believe every individual has a personal experience which is different is there any other thing you would like to add on your experience.**

P4: For me my experience with CARTA has been a great one it has been one that is very positive just like my colleagues said I am also I started the program like I started the program I was on a PhD program before the CARTA fellow ship came in and I knew how I struggled to carry out the projects, the research because my research is more laboratory based I needed a lot of equipment’s to procure and buy reagents and it was difficult one but with CARTA I was able to access the research funds and I was able to acquire those equipment to carry out my research and I think the finance CARTA has been quite helpful. And also in the area of bring like minds together and you are able to rub minds and learn from each other you learn from your colleagues the ability to learn from peers and you also see what others are doing and you also have confidence that yah you are also doing well you can listen from other people are able to see the kind of research they carry out the way you think the way you do things changes so CARTA has been very positive in a way.

**Mod: Is there anything you would like to add on that.**

P5: Yah sure in my opinion I think CARTA has been a catalyst of change among intellectuals in terms of you know as intellectuals coming from teaching in the universities I mean we were in our comfort zones and so actually CARTA pulled us out from that comfort zone putting you somewhere where you are made to learn from your fellow friends having different backgrounds and that actually to me meant quite a lot , but again my experience has been that CARTA encourages multidisciplinary kind of PhD programing and that actually is good because I don’t think I have read any other program initiated in Africa that actually encourages multidisciplinary approach in term of doing research but again I see to it that that is on the positive side now , but on the negative side they are issues must be looked at seriously , my friend talked about the issue of the focal persons that we have in our institutions I don’t want to talk for one university but within CARTA initially we had a number of universities but as am talking now we have one university that pulled out now I mean for the sake of because if we go ourselves as researchers but one other thing I don’t know but there was need to actually to tell us or just to inform us that such and such a university has pulled out because of such a reason because we may not want us as we move out to leading positions in our universities to repeat the same kind of mistakes, but again I think there has been other issues I know CARTA is an African program we are in a consortium of about seven or eight universities and you find yes we are allowed to be to move from one country to another to carry out your PhD but I can think that sometimes it pushes people to shame away from their own universities and at the end of the day you need to sell your own university but any way there are opportunity that you can go and do your study with a university outside. But again in line with that you find that they are other issues that are not clear with in our universities, I will give an example of course universities have different policies with the way they run their affairs but you will find that for you to do such training with your own university some institutions requires you to pay some fees and so at the end of the day what does that tell you, it means if I have got this PhD opportunity then I am pushed out of my country to do it because if I do that then CARTA pays for my tuition fees much as for me I will want to seek CARTA I mean it should have a certain percentage of the students to say that if we have enrolled five from my university at least a certain percentage should enroll from that university because if those people of that place cannot showcase how good their universities are who can do that, so I would want to see CARTA or propose or suggest if we have enrolled may be six students or four under this university to see that certain percentage can enroll in their own universities so that we are able to showcase how good because I don’t think there is no university that would not want that I think those are the two points that I have to share so experience to wrap it all it has been good and we want CARTA to keep up the good work .

**Mod: okay please**

P1: I think my colleague here is touching on something that is very it is a grey area I think that why he said our focal persons need to step up also like in my own university there was that issue and when I raised it to the focal person actually in fact he did not do anything and I took it upon myself I said you know what no this is what they signed for and therefore they should wave my fees I had to make sure that they did wave for the first two years they did that but . They brought back all the fees for the last two years and then I am like you know what I told them that if CARTA can’t pay all the fees and I register in my own institution but then I said there was no way this statement is going to be sent to me every month so I wrote to CARTA straight and they paid all the fees including even the year that is the subsequent year that I was going that was to come so I felt that those funds that are within our universities they had deposited so it was the focal person to know that use this course to charge for another course it can’t be free but they have to waive it so when they waive it has to come from some source within our university but they had no clue so we had to investigate for them so I wrote straight to CARTA and CARTA paid them and that was the end of the story, and also I think also one thing just I mean just something we were discussing with the colleague that are some universities that they put you on three months contract then after three months they renew so that thing is like a revolving structure so I am saying they change, so are the focal persons saying CARTA is training under CARTA academic because if you on three months six months one year what makes them do that they should make it clear to the focal person that for CARTA with this university we are not going to accept this because you under this academics, I think that also needs to be waved off and clarified that these institutions that they are signing know what they are signing for we don’t want these institutions that will just put signatures you know that annoys a lot.

**Mod: Our next guiding question I know we have already hinted on it as people who have been on CARTA program how has it impacted on your PhD study research world, relationship with other researchers**

P: (.) could you repeat the question.

**Mod: How has CARTA impacted on different aspects like your PhD journey though some you had already hinted on it, research skills development, and other relationship with other researchers in your home universities or other researchers in the world and your view about the research world?**

P3: In terms of the research skills I will talk more on the research skill then I think CARTA on these JASes in which we are always involved they are enriching because of the PhD in our universities which we know most of it is not structured so the moment you enroll there is no any learning apart from you and your supervisor doing this work so while CARTA brings in these JASes in form of seminars of which they are tailored to enrich the research skills like the first JAS is about epidemiology and other research skills when you are going to the second one they are now enhancing your analysis skills by such they literally enhance the person in the normal way unlike majority of the programs in our countries , sometimes they may have the programs where they may have the program where you have to fund yourself while for this one it is embedded into CARTA , the research skills were being enhanced , the relationship with other researchers yes we are leading into the system, it depends where you are working because sometimes you can have a paper, it depends on the department you are working how are they in line with your field so some of us we may not say we have enhanced this so we can interact with other researchers , but completely saying we are interacting with other researchers completely may not be so because we have completely different fields and we feel collaboration in with in so that will depend while other people may be in a field where they can collaborate because their departments are wide enough so it depends. Also, if so that may be more subjective but so as for me that may not be slightly much as for the international [ as in the research world how do you view it] I think I will comment later for the last one it is hazy.

**Mod: What of the rest of you how has CARTA impacted on your PhD Journey, research skills, relationship with other researchers, and how you view the research world**

P5: I think for me well I have been in the research field for some time but being part of CARTA it has actually strengthened me in a way I need to conduct research because you know sometimes I think initially, I talked about that at times you do your own things in your comfort zone and you feel that everything is okay about you are doing things but having been exposed to this kind of training where you have met different people with different experiences your own fellows from different universities with different experiences that actually made me to look at myself as someone always who can learn from others because you know research is not a one off thing or it is something that you might think because that because it is working in Malawi that it can as well work in Nigeria but you need to listen to others so through the CARTA training in my opinion I have been able to learn from others I will give an example of the research Topic that I have now it is the only one that I initially thought I could do but by the fact that I was brought into this school and am listening and asking the colleagues, you find that it actually improved the area that I was focusing and so much so that with that kind of interaction it has helped me to refocus in my area of research but again beyond that you find that I know for sure that if I am to do a particular research or if I am to venture into a particular research area I think I can always ask this other colleague may be to give more views on how best I can do it so that kind of net working within ourselves to me I can see that of all I have seen immediate fruits out of that , but I can fore see or visage fruits that will come out of that in the future , you have asked issues with any international…

**Mod: How do you feel about the research world?**

P5: The research world or the research work?

**Mod: Research world, in the world where research are being carried out**

P5: I don’t know if I am answering directly your question but I think just being part of CARTA I have directly or indirectly been connected to people internationally so I see to it that when I want to venture into a particular research area there are people that can help but beyond that I think through CARTA I should be able to and actually if somebody hears that I was with CARTA and I write for a grant it has actually put my CV something internationally, So to me even if it has not happened but I can feel it has happened the impact which God willing I will use it for life.

**Mod: Are you eager, motivated to go into the research world having been part of CARTA?**

P5: I would say yes but I don’t know if this will come later you know being in CARTA I have not signed a bond so I might decide the moment I get my certificate I will go to Geneva and I am venturing into now because of that I would think that if may be CARTA would be having something like a bond like you guys we have a bond our main focus for you guys was for you guys to go back to your universities and institutionalize whatever we have imparted upon you but because there is no that kind of bond so I may decide to go home after my PhD and decide to go into politics and become a politician and get a lot of money having struggled for four years of CARTA with 20% cut of salary within your university, so such kind of things, so yes I think CARTA has played a role on where internationally I can do research. I don’t know if I have answered your question.

P1: I think for me skill wise I think I have benefited because I trained on qualitative research methods but with CARTA I have learnt a lot of quantitative approaches of research and I have also with researchers I have collaborated with two or three researchers from other universities , they are few papers out there on review so it has sort of linked me to people outside my field being interdisciplinary my colleague is talking about here and then in view of the research world I can see that everything is possible and when I look at my PhD now this was like the beginning of work so I think it has been very good. I have gained in form so I begin to say that yes this I can use now, so I think it has been a very good kind of. I think I have gained a lot in terms of quantitative as I was saying so it has been of help.

**Mod: Okay is there anything you would like to share with us about your teaching skills add on your view about leadership skills**.

P2: Before the teaching skills Let me add mine on the research and research world I think they were something that is completely different I would like to build up on what Lesta (all laugh) what my colleague has said what the two participants I will also say first of all I will talk about the PhD research world and research experience I will bond that together that before I came to CARTA I used to conduct research as if it was a big walk really coming to CARTA it made it like a research can be found to do it’s kind of sort of made me losing hope and see it in a different and different perspective completely it has encouraged a lot of collaboration a part as my colleague has said couple of things some research work having started I remember recently before I joined CARTA there used to be a problem when I am submitting manuscripts in a number of journals then they would ask me to suggest reviewers then I would say whom would I suggest but right now it is just easy to say I can just look into my cohort and say hey you review for me, and I think that just makes it too much that even when I am calling colleagues to review for me I know the colleague is going to give a very critical and very you know they would give quality review I think with that CARTA has made a lot of things to reach out, when I am looking at which part of Africa what research we can do in regards to which person can assist me in that kind of research enable and I think also I would like to bother on what the first participant said concerning the bond and in terms of research world I would like to say that is not much of the positive the fact that the bond we signed wants us to go us back to our institution , I think it is a kind of limitation because if you get a job opportunity in WHO or somewhere bigger you are still held to that bond that I have to go back to my home institution I think CARTA needs to broaden in that area that you can step on bigger places and not just limit you to your home institution I think that needs to be reviewed that is my view on the research world I think the last one which was [ in the research world having been in CARTA I think you have hinted on it] program have you been motivated to , but I just think the limitation where you go after CARTA should be little bit reduced in terms to just you don’t have to just go to your own institution.

**Mod: could you share with us okay?**

P4: okay just to build on what others have said my skills have greatly been enhanced through the CARTA journey in away the jases have been structured from jase 1to jase 2 ,3 and 4 they have greatly helped me to in various ways they for example in jase one where we were taught various things like how to write our paragraphs and before you would just write anything and you think it is fine but now you have to be conscious like okay just the paragraph is addressing a particular issue so the way I write my manual script has just greatly improved that is why I think CARTA has played a great role and also the issue of critical thinking the way you think so for example when somebody gives you a proposal to read and you think in a way you will be able to make comments and everything without you having the idea of what the topic was before you can heavily contribute to that critical thinking and also the issue of the kind of places where you publish your journal so before CARTA I could just publish my paper anywhere my manuscript there is like there is a standard that you don’t want to go you are inspired so CARTA has built that confidence that our manual script can be accepted in top journals so I think CARTA has done a lot. And also many journals in terms of collaboration and everything in our previous schools we have colleagues that we are writing manuscripts together in the previous cohorts so the issue of CARTA bringing us together to do things together it is positive.

**Mod: Still as we are still talking about how CARTA has impacted on you I would like to know how CARTA has impacted on your teaching skills , leaders ship roles and your understanding about mentoring**.

P2: For me in my institution what we used to understand while teaching was stand in front of the class and just talk and talk and talk for two hours and you find everyone is sleeping as you are talking that used to be my impression of teaching before by then like a bondage of sleep but coming to CARTA changed my orientation that there are different ways in which you can engage people to keep them active for two hours you walk around , breaking into group discussions using nice innovational technologies use of video clips use of all that as a matter of fact that there is no class that I teach right now that I don’t put in video class/video clip and some people have started saying that ooh I like that guys class we gonna watch a video and love it so I think it has helped my teaching skill now I don’t have to talk for two hours I know some of us come from institutions where you teach a class back to back it can actually be very stressful when you talk for two hours but now I take classes back to back because I know half of the class I leave them to engage themselves and the end of the day everybody is happy they go home refresh they go that teaching experience I think CARTA has put a lot on my teaching skills that are so diverse in many ways and those are the things we have been putting to skills in my own institution .

Mod**: The gentle man in white are you with us as if you are not following [yes am following] can you share with us how CARTA has impacted on your teaching skills understanding about mentoring and the leadership**.

P3: That it means they may different views on leadership and mentoring so it depends on the different fields some of which may be different so I have had a good supervisor who has been mentoring me so since had good supervisors whenever I would do anything I would do the right thing even for the proposal, getting a grant do it together the moment we are here being given the guide he would swap my name and put his we have the client so they would say no this is the one to investigate at the same time CARTA has taught me now how to mentor now because by then it was mentoring under a supervisor but now we made progress in that we are supposed to mentor other students who will be coming as for teaching I have included different things whenever I am teaching some initially some of the things that I was doing mainly was giving assignments usually to students requiring them to use standardized way of referencing which initially there was no much emphasis the issue of plagiarism and writing skills of the different students and continued giving them assignments and whenever I am marking them so when I look at their assignments I tell them this ids the standard to avoid plagiarism so that has really improved the teaching skills .

**Mod: Is there anything you would like to add CARTA has been a journey that has had its low moments and high moments, could you please describe what you consider to be your low moment and also describe your low moments.**

P4: For me I don’t think I have a low moment in CARTA as a journey my high moment the high moment was the day when I got the mail that I was selected for CARTA I was so excited because the previous year that was my second attempt the first attempt we applied with some of my colleagues and we were denied and I was somehow rejected because I was looking forward to peruse my PhD and I will be able to get my everything I needed but for this time around when I got it I was excited that was the high moment for me.

**Mod: so you have not had any low moment in your CARTA journey.**

P:5: no low moments.

**Mod: Others yes please.**

P2: My journey also in a way starts like for my friend it started with low moment and then a high moment low moments then high moments (all laugh) and I will go through them one by one so the low moment of course was also I was once rejected I felt it was the end of the world I really felt there was no hope for ever for me so it was very horrible time for me thank God I had people in CARTA who said you know don’t give up I was I did not give up I think the comments I got from the first application were very kind of harsh they were very harsh and discouraging I felt I was the dullest person that was moving around I felt I was that there was no hope for me with those comments you know but encouragement from other CARTA fellows made me know that if I applied again it there is something good that will come out of it and of course , so as the other fellow said when I got the email too I was so happy that I could not almost not drive up to home I had to tell somebody you know come and drop me home because I did not know what I could do ( all laugh) so that was the high moment regards to me now the next low moment is when we got to jas one this was another thing that brought a low moment for me and I think in jas I streesed one thing I don’t know if it has been looked into now when everybody was coming into Jas one the impression was that everybody was coming with an institution and a supervisor and they were just refining proposals and where most of us were abandoning our institutions and looking at other institutions so it was like a transition period for some of us so for me in jas one it was a low moment when I found out in second week and third week when I was applying to it when they told me no CARTA has nothing to do with that you find your school you find your supervisor ,so for me it was a very low moment by the end of third week , by the fourth week when everybody was settled with their proposals I was nowhere and nobody was listening to me I need a school how is CARTA going to help me get a school so I felt for me that was a low moment and I think that should be looked into in jas one especially when people are coming in I think they should be quick to say you guys who have schools come this side , you guys who have no school come to this school let’s see how to help you people should not be left at that stage to fight for themselves to sort themselves out so I think with that it actually set me back by one year so as I talk now am actually in my fourth year of my of CARTA fellowship but I am in my third year of my PhD program because for the whole entire of the first year I did not get a school I had to keep sending emails going back and forth I was getting rejections there was no assistance for me anywhere, until later they called director of studies and they helped me of course all that time she was in school she could not release out so I think that when she stepped in that helped me I was able to get a school in my second year of CARTA so for me at that period of time that was another low moment yah I was having the normal CARTA support and all that but for me my main interest my main wish and my main help I needed at that time it was a school and I did not feel it was answered on time so I think CARTA needs to look into this and of course every other thing has been positive otherwise I got a school everything has been smooth I wouldn’t want to bother you with all get all the information of course they were moments we would get dreadlocks CARTA would come in CARTA has got those privileges to help people overcome those dreadlocks so those are basically my high and low points.

Mod: **Others could you share with us your low and high moment.**

P1: I think for me the low moment was during my application period when I applied they were some colleagues who were rejected during the previous years so when I told people that I saw this advert and I wanted to apply they were like no you will never get into that journey by the way all those negative sorts of things I was like wow and when I was applying I did not tell anyone that I was going on with the application and yes I applied but I had that doubt that no one gets it if this one did not get it do I have the chance but came October I got that email that congratulation that you have got it that was a high moment I could fore see that this PhD that there is life at end of the tunnel ,now that I did not have to bother anyone with funding and those were the high moments but when it came to the school again people could not believe it is like they still had that but how and I said this is how I did and anyone who wants to apply can get assistance from me I did not know that there could be jealousy in this kind of grudge because some of these guys thought how could this guy go through I couldn’t know that this could happen in academia because I think in academia were thinking differently but when I saw that I was like I have to be careful I achieving and completing definitely that has been the high moment and ever I could pray that CARTA could how one feels and how this is the program that takes a credit for this .

**Mod: what do others have to share with us on their low and high moments with CARTA?**

P5: In terms of I think most of the people have shared on the high moments, I think they are times when you could really feel that you are down and you are like do I see myself out of this PhD program. I will talk in terms of number one I think they are issues that happen with in institutions, without naming institutions we have had issues where administratively you have been offered this kind of fellowship scholarship but you find it takes long for someone to just sign on documents and it goes back to the issue of a focal person so you ask a focal person there is this issue this person cannot sign this then the focal person says that no you know the position I have I cannot influence this guy to complete this kind of thing so actually I would give an example that my institution actually took almost ten months before someone could just endorse not knowing that someone was just keeping the documents, so actually you have been awarded this high important kind of fellowship but somehow somebody is not refusing but he does not want to do the work so that is one of the low moment that I went through . But again you find that as you have started the program you find that the supervisor supervise relationship is as if you are in a competition and you say may be that how all PhD is all about, but I would want to see it to that such moments should not be there but overall I thing being part of CARTA it has been full of high moments but with some moments when you feel low and you feel you have been reduced to zero and but things have been going on.

**Mod: When was that time when you felt that you had been reduced to Zero is it when it came to the supervisor supervise the moments when you really felt that you are low.**

P5: Well I think they are moments whereby I think somebody has talked about the issue of mentorship where we always assume that somebody is a professor is a mentor the fact that they have studied quite lot and they have been promoted through the ranks you would find that they do not understand the relevance and their role in terms of mentorship, now you are there not to bother them but they are there to mentor you so that you can be to a level where you can be able to take yourself as a researcher and even lead national and international research so these are some of the moments that you see need help from your from your supervisor but you don’t get the actual help from your supervisor that you should be provided so those are some of the moments that you are really low but a apart from that may be that is how life goes .

**Mod: You have shared with us a number of challenges that you have encountered in the course of this fellowship is there any other challenge you would like to share with us.**

P3:I think the other thing is the schedules or time table for the jases sometimes they are not conducive for our PhDs [ so what would you recommend] for any jas there should be time for individual work whether it is four hours in a week but when you have a lot of assignments most likely to do something for your PhD else assignment after assignment there for by the way at times when we are doing jases sometimes we stop working on the PhD work sometimes people go there and say I will go there and do my work a,b,c but we are here overloaded with information not living a spare of two hours in a week or may be two days in a week that would make people really work that is my comment.

P1: To add on what my fellow colleague is saying I think those jases really… have to be restructured , really need to be strong attention to it and the reason why I say that is that every jas people are coming from different countries and then the second year people are assembling in class by eight o’clock and they are slumbering until six and some people have not yet even called their families to get new local simcard to go and shop I think the first day should be left alone to or half of the day may be the classes should start at 12:00pm so that every morning people can sort themselves may be buy simcard and also buy everything and also I think just starting at 8;00 o’clock everyday people are moved by 7:30 I think that is quite too early I think I am not trying to sound lazy but they are some people that don’t have those early mornings I think if normal jases start at around nine and probably extend it to five it could allow some people wake up early get themselves ready before they slum themselves into class and like the way my friend said even when you are doing this there should be some time to do your personal work not like back to back lectures where you get to six and you are back in the evening and you are almost burnt out I think the productive hours is really that between nine and four so by six you are tired and you getting back you know I think that should be looked into the timing when the jases the lectures start and the time you are resting I think they should review it.

**Mod: I think most of this contribution you sharing are rotating around the jases and I understand you had different jases the first one that was in Nairobi , you had another one in South Africa ,then Nigeria and this current one in Uganda and you had a number of activities.so on top of the recommendations that you have shared with us what do you feel should be done differently to improve on these jases in terms of funding , internship , facilitators selection , a accommodation , program schedule that you have already hinted on , assignments then training on scientific writing ,payments and deliverables I know it is long but I will always repeat as we discuss.**

P2: One thing I have picked out is the area of the facilitators I think some facilitators need to be reviewed , I think some people need to be dropped and some people should be encouraged to come more and I think the facilitators brought to jases need to be evaluated and I think there should be a secret ballot box in which fellows should be allowed to comment anonymously about some facilitators and I say that because of my experience in jas three I got paired with a facilitator that almost spent two hours bragging to me all where he has published and could not offer me any help concerning my analysis and I could not dis engage myself and I was there talking with him for two hours so that kind of thing so some facilitators in jases should not be brought so that the fellows can make something good of their time to be well planned out.

P3: Then for anonymity they are some jases where you need to give your views on line but there should be anonymity some of us were not honest enough because you find when you don’t give a feedback they say you have not said anything that means somebody knows[some body knows] this person has not done[has not commented ]then the result so I said for these one I will keep un honest and I give a fake opinion [ so what my colleague is saying that the emails are not anonymous enough , evaluation on line is not anonymous ] there should be away to review that so that people can actually be able to give very honest comments without being twisted without it being linked to them.

P4: I also want to add to that you find out that they are some course schedules that the way jas instructors allocated a lot of time to particular topics and you find such topics are not all that relevant compared to some that are so relevant and they are giving them small duration for them for example there was a course that we did today the one on slides I think the man was rushing , the man was really rushing because I think the time allocated to him was so little compared and you find out in some days some of the things we are doing do not make sense and the time allocated to them is a lot so I think the time allocated to them should be looked into so that the ones that will benefit the fellows should be given more time too

**Mod: How can this be done for the fellows to benefit from all they have been given in relation to the time allocated to the different courses.**

P2: I think in every jas they should put suggestion boxes so that the fellows can evaluate and put it there without their names online can reduce and be twisted so that is also effective all of us can just can decide to write what is nice so that the guy on the other side can say that this guy is a good boy when there is a box right there at the corner we can always go and say that ooh my God this session like this session needs to be cut down for example the session my friend is talking about we could have been desirable if could have had hands on not just rushing in 10minutes hands on and that take we would benefit from it and the things that man was saying were skills that we all needed but he was just training us in power point and we couldn’t know how do we do it [ and we couldn’t just divide into groups just talking to ourselves it couldn’t make sense we really needed hands on ] I think a suggestion box is needed it is very conventional and old fashioned as people are trying to move to the technology world but some can’t be taken from conventional talk a suggestion box is very anonymous and I think it would work it to get some good feedback .

P5: I think what is being stressed here is the importance of evaluating the topics that we are given to go through so as much as for us we may not experience that change of things but we want the colleagues that are coming after us actually to have that as they are saying we really find that they are some topics that are taken through us but at the end of the day you say but was that relevant of course it might have been relevant to someone but if you have evaluated that particular session that evaluation I mean would be objective views would be coming from different people they would rate and it would be of help other than just rushing through the things and repeating the same things year after year the same facilitators year after year so evaluation very important .

P1:I think the other thing we may want to look at is that from Jas three to jas four is plus seven or minus eight months so may be if they can if they can think of the assignments allocated especially teachers of because if we have an assignment after an assignment then maybe you are trying to complete you are writing a final chapters you are getting feedback for that so you are left absolutely with almost no time to finish that and sometimes you are at the pick of writing and you go down at times you go two to three weeks four weeks without touching on your PhD that means when you go to it you know that the pick that you might have developed might have lowered I think that consideration and if may be they are to be assignments or something like hands on as my colleague was saying from 8:30am to 5:30pm honestly there is no enough time to kind of that there is no enough time to do what you can do so that when you get to the room people sort of focus on things that are constructive and may be that need to be improved jas three we are writing there is nothing jas two you are also there jas one you it is all most losing value so people might need relieve it is something pushing there but these assignments .

**Mod: you have shared with us about assignments, facilitators and the program schedule the time given to each area, is there anything to add on funding, internship, accommodation and payments on stipends and deliverables you had to submit in as Jases 1, 2, 3 and 4**

P5: I think there is an issue about money, not the money I get as stipends but research money. I should suggest that CARTA needs to do be flexible because there some other research that require little/less money but they are others researches that are vigorous that they need you to buy reagents and all these other kind of things but CARTA is not flexible but yes for those who do research and do require a lot of money its fine but those who need expensive gadgets to run samples should be considered because it’s not everybody who can go to the institution and say can I be assisted with $3,000 that they would be given or people might try to apply for external funding from other institutions and they were not successful. So I would suggested that research money may be if CARTA would be flexible in terms of allocating research money for those that requires a lot of money provide them we know that it is seed money but it must be flexible according to research categories.

P2**:** To add on Research money I need that I want to state something that CARTA is doing that is very ,very good, the fact that it stipends are dropped directly on CARTA fellows phones or account I think it’s a very big loss because we know institutions have bureaucracy and when it gets to the institution it might be the end of it, it may not get to the CARTA fellow but think that is a positive thing that CARTA is doing is good and positive strong point because I have seen a lot of scholarships where a lot of people are stranded on the programs because the money gets stuck with bureaucratic organizations who want to sit on the money, and I think that is a very big loss. I think we should also review the research inter jase deliverables I think they were nice when I was in jas four I felt the impression they were very serious about it and paid so much attention on it and found out in one of my reviews and I now got a feedback that I wasn’t serious because I was focusing on jase deliverables not focusing on my PhD so for me that was a total confusion because you tell me to have inter jase deliverables and then come back and say I should focus on the inter jase and not my PhD. So I think there should be a balance on that because what is willing or what is expected of us in terms of inter jas deliverables , I think it is important doesn’t matter if we don’t meet up with them, doesn’t it matter . I think those should be looked into those are the two things I felt like sharing as we were talking and I felt I should bring them.

P1: I think in terms of jas deliverables I am not sure how they are doing it in jas one , two you know at some point I ask my self does CARTA know that I still work here (laughs) you know assignment in jas one you know you had to do because that was a ticket to jas two but that was quite a lot and I think and may be during that time if they wanted people to do it they should have for your teaching I don’t know if that is still the case that is the only thing I failed to know in terms of deliverables it needed some review because one thing when someone is waiting for an assignment yet you have deliverables, a class to teach and tutors to supervise, then the PhD and a presentation. So it was quite a lot of things and at the end of the day I think a lot of people they you tell people about CARTA and you talk about the other guys then the other two it was a lot and they needed to look into in terms of deliverables.

**Mod: okay, about the training on scientific writing is there anything you like to be done differently?**

P2: Well training on scientific writing what I can suggest if there could also be something like the structure of jase if possible so that people can be brought together sometime to have a writing time, some institutions have a writing time but I don’t know how financially the complications will be for CARTA which could be done for a week or two weeks where people come and meet once in a while to have writing time on scientific or manuscript writing I think that will be very good.

**Mod: on accommodation what would you like to be done or improved on?**

P1: (laughs) I think to me on accommodation with jas three am not sure if they have done anything on that CARTA I don’t know if they have done something with CARTA hostels, the planning was fine but they needed to open up those things and add communal toilets then open. But this is the worst accommodation I have ever had, when we got in as soon as after painting the ventilation is poor you can imagine, the paint is smelling although we struggled. So I just hope something has been done with CARTA hostel not so big but we need something that is conducive writing because we can’t be in an environment that has no lighting and we were forced to sit there the whole day place was kind of small. Literally some people like their space even when they are writing either in the library or your room but we had no choice. We know they were trying to finish the work before people come in but they would find other alternatives before.

P2: I want to add on that when you mentioned about accommodation in Nigeria but never wanted to talk about it in a different place because it is my country but the things CARTA is trying to manage there was initially another institute in Nigeria managing which was Tropical Agriculture but I don’t know why they moved from there but I think that place could be a lot more conducive for learning in jas 3 considering the writing a cross jas 3, if CARTA could consider using that institution it could go a long way in improving the productivity in jas 3.

**Mod: as we come to the end as CARTA fellows where do you see yourself in the next 5-10years?**

P5:(all laugh) where do I see myself in the next 5-10years, life is unpredictable but I want to see myself as a research leader because I mentioned this at the beginning but the challenge is CARTA has invested in me through my institution but there is nothing that will restrict me when I go back and became a good researcher but the next question will be where? I will go where I get a lot of money but I will think about what I will do for my own institution, okay assuming tomorrow CARTA is not there I should be my own CARTA in my institution and also implement the things CARETA has taught me to be an international researcher.

**Mod: okay, gentlemen where do you see yourselves in the next 5- 10years?**

P4: just to add on what my colleague has said, in the next five years I want to see myself as a leader in my research field (laughs) I want to see my self as the first in my area of research if possible(laughs) my friends were saying something we will be prof in the next five years God willing.

P1: I was just looking for a picture here in my phone that shows a modern day track that they use in the United Kingdom and the normal track we use, so the modern day track is a link to see the day I started with CARTA proposal which is a very nice one but now completion is like this other tracker which cannot even tell if it can even move or reached so it’s like we have gone from riches to poverty. In the next 5 years I want to rebuild my lost empire staff and then consider my research leader in a particular institution but I feel CARTA has given me the tools and it is my time to correct whatever went wrong in the last 4-5 years.( shows the picture)

**Mod: could you please share with us the picture?**

P1: yes, this is a very good picture [it would be used to give a good report] my phone memory is small but let me forward it to my colleague [do you mind him forwarding you the pictures and I will get it from you please]

P2: I think talking about myself where I will be in the next 5-10years I will follow my need by composition and by the fact that I signed a contract with CARTA to return to my own institution. I see myself as a new person discharging and imparting what CARTA deposited in me in the younger generation and that will be done in the next five years with that CARTA has bonded me I don’t see myself like someone who loves teaching so I will run and move into a research organization probably an international where my research will be translated into policy and action that’s where I see may self in 10years now not

**Mod: gentleman in white where do you see yourself in the coming 5-10years?**

P3: I think I might be an outliner [why?] because as of now am more involved in discovering the engagements in the flow and review of the laws technically in academia. I don’t want to go into administration or for higher positions in the government, that’s why I will be an outlier because of my freedom of movement without that I was supposed to go into administration but still I do government when they need me like last year I was so much involved in government activities so it is hard to project [so it will still be academia] I will remain in academia.

**Mod: in regards to academia will go further more in research or?**

P3: As of now I have developed have a research portfolio what I have to do is expanding and employ people so as to build the capacity in my PhD program so we are still building capacity in my area that am doing my PhD because it is more of research information we have a funder who funds us throughout but even if they’re not there but thinking is after several years it will be a platform for my thinking after several years being a platform applying directly with the European funding’s so I am still building capacity so that within 2-3 years I can directly establish the portfolio and I will be getting funds directly from that portfolio .

**Mod: okay, thank you very much we are coming to the end of this discussion if there is anything you would like to add on CARTA fellowship before we end?**

P2: let me say I would like to not act like Oliver twist for the sake of the new people coming in, it will be nice if stipends for can be reviewed I know CARTA fellowship is doing so well right now but I know a lot of more coming in will need more funding and can be done better .

**Mod: okay thanks for your time we have come to the end of our discussion.**

**Thank you for your time**
