# Supplementary material for: Experiences of doctoral students enrolled in a research fellowship program to support doctoral training in Africa (2014 to 2018): The Consortium for Advanced Research Training in Africa odyssey
Source: PLoS One. 2021 Jun 10;16(6):e0252863. doi: 10.1371/journal.pone.0252863 (PMC8191976; doi:10.1371/journal.pone.0252863)
Supplement: S2 Transcript — (DOCX) [file pone.0252863.s002.docx]

**CARTA FGD**

**DATE OF INTERVIEW:** 12^th^.03.2018

**CATEGORY OF FGD:** Females only

**MODERATOR:** Robinah

**NOTE TAKER:** Resty

**TRANSCRIBER**: Resty

**NUMBER OF RESPONDENTS:** 07

**DURATION OF INTERVIEW:** 1hr: 22 minutes

**KEY**

**MOD:** Moderator

**NT**: Note taker

**P1, P2, 3**… : Participants

**Mod: our first driving question will be. Could you please share with us your experiences with CARTA? Each of you can share with us your experience before you could share the general experience.**

*Silence*

**Mod: and we do not have a beginning point any one can…**

*[Laughter for all]*

P7: I can start by saying the experience with CARTA has been very great in relation to research. When I was joining CARTA my knowledge and skills in research were at a lower level but then I can see myself that I have moved from that level to another level. And again what I like with CARTA is the combination, training you in research and again as an educator and how do you teach? So those are some of the things which I can remember CARTA with.

**Mod: how about the other ladies what do you have to say about your experience with CARTA?**

P5: For me my experience with CARTA has been on the positive side and I will say that I have benefited so much from CARTA Training in terms of my teaching skills and most importantly my research skills. And I will also agree with the fellow participant that CARTA has really impacted greatly on our research skills and I think everybody will agree with that. And also the way, when you come to interpersonal relationships I have also been able to learn and because we have diverse discipline and culture and the multidisciplinary approach we really learn quickly and we see things from different perspectives. That also really helped. And it has also improved my how to present information, how to relate with people and also collaboration.

**Mod: I think about having improved your research skills, can you elaborate more like could you compare what it was before you joined CARTA?**

P5: okay, so let me do it on a scale of one to ten [1-10] before I joined CARTA maybe am – especially with data analysis and report writing maybe I will be at four but now I can say am at eight[8], so that increased.

P1: for me it’s been a journey if I could say so, a journey that begun with little steps and now making new big steps if I can say so. It started with people that I didn’t know, we started virtually I was like ‘’ohhh who is this you know fellow from South Africa?’’ Say, when you need to work on assignments as groups, you have to do it as a team we got to meet each other in Nairobi and wow I was like okay. You know the fact that we had been told that you are the cream of the cream we made it as twenty five people [25] out of the very many that started made you feel like yeah I can be able to work through it but then in the middle of the way you get lost, so I got lost but I found my way through some of the JASes and to where we are right now its effective or rather having not completed our PHD at this point I think it’s been something that has taught me you know the PHD process. The people who are saying they are doing PHDs they are work through it and you never see how that is done until you actually experience it yourself. So I can say it’s been a journey for me.

**Mod: okay I guess everyone has had an experience.**

**[Laughter]**

P3: For me also it was a journey that I started and CARTA is a unique kind of collaboration because it makes you have hands on learning and before we even started our JASes we had an opportunity to work with the team online and we would just see names so honestly there are some names I saw and I thought they were male so I would see xxx and I say yeah this must be a male from South Africa now when we met in Nairobi I was shocked I said haa this is a female.

So honestly in a scale of one to ten I would say when I began I was around three there and now am climbing to six there so it is a process and as my colleague, and my fellow participant have said when you begin you know you are thinking you are on top of everything you are thinking ohhh am in CARTA you know you are walking around in the corridors of the institution everybody says congratulations you know I made it over two hundred people applied and only twenty five people were picked’’, when you start the training challenges begin. When you go to class you say ‘’ohhh I used to think I knew’’, personally like things to do with proposal I used to imagine I knew until I went to class and found out that it was a bit – you get totally lost but good enough it’s the only – for me it’s the only collaboration I have experienced where they bring people from different areas, facilitators from different institutions far and wide and they help you to walk with you to where we are so we are grateful.

**NT: maybe just one more thing, can you speak a little bit louder so that we can be captured on these recorders, so that when we go back we get everything clearly the way it is….thank you.**

P2: For me CARTA has been quite an experience, it has changed the way I think, the way I look at things. Okay before I joined CARTA I used to think of research in a straight way just in my field, I didn’t think of it beyond - but this multidisciplinary brings people together you now see ohhh this is how a mathematician fits into my thinking into my line of research, this is how a social scientist fits in, this is how, so it has expanded my thinking of research.

It has also helped improve my research skills, again from a scale from very low level to a higher level.

Then the platform the way you are able to interact with people from other countries, we have a saying in my community that; ‘ a child who doesn’t go out thinks it is only the mother who knows how to cook’ so after applying this situation you are able to see how things are done in this other country. So it has been a great experience, so the fact that now I know them you find that a particular University has rights to say journal articles which your country doesn’t have in the University and because of this we are able to share that kind of info, to get access to such resources that without CARTA probably we wouldn’t have gotten.

Also my teaching skills have been shaped differently by CARTA, I would say I have improved the way I used to teach before CARTA and now. Yeah.

P4: Okay in my case I conquer with my colleagues I have improved in many aspects I will start with research wise I remember thinking that you know sometimes when you have gone through teaching research for a while you think you know, in fact it made me realize that one can never learn enough there are always new things to learn so I have gained a lot in terms of research and focusing on my topic. I have worked internationally and mostly you know in my institution I had never been able to interact with other countries, so it was an eye opener in fact that made me appreciate, it was a humbling experience honestly and to able to see the breath and the depth of informational knowledge from the colleagues. In terms of teaching I thought that was my strength, I learnt a lot, I gained a lot from the approaches and but I also learnt, I like to call it a soft skill you know just being able to cope to manage sometimes you think you have issues only to hear other peoples’ issues or you think your issues are small you know it is just to relate and then it focuses your problems emotionally and it gives you strength and endurance to go on. So relating with other colleagues and I must say many people from my country we tend not to travel, at least, at times, we think Africa so I learnt a lot from the great personalities we have in the country, so it enriched me that way.

P6: So I will describe my journey with CARTA as awesome and exciting I actually I said I wanted to do a PHD not just a regular PhD but a high quality PHD. I think CARTA has given me more than I actually bargained for, I have sharpened my research skills, for example I had never done qualitative research in my life, and in my PHD I am doing mixed study research. And even in my university I have been invited on different fora to come and teach qualitative research. So I was like wow; how many years have transpired and am now teaching people? You know even research wise we have been given the opportunity to come and teach people.

Not only that, i now appreciate the value of other staff on the research team like the administrative staff, I have learnt a lot about how to organize research teams. And CARTA has also helped me as a person to like develop my career area give myself time line, working on the projects, publishing, I will say the effect is indescribable.

**Mod: Okay thank you very much for sharing with us all your experiences, our next guiding question some of you have already hinted on it on your journey how CARTA has impacted on your different areas, in skills in teaching, skills in research but we can go back to it and get more in-depth understanding of it.**

**So I would like each of you to share with us how CARTA has impacted on for example the entire PHD journey; research skills, relationship with other researchers in the world and your view about the research world we begin with those.**

P4: Okay I know we have all been positive in saying all the good things but in terms of my personal experience in my field or my discipline I was frustrated in JAS1; when I really needed guidance in terms of starting in other terms in addition to knowing the general stuff you know the overall topic, the conceptual framework there was a time when I needed technical input in terms of my field, so the facilitators they attempted to help me but by week three I was very frustrated but fortunately because of the partnership with other partners I managed to get advice. So, yes it has been good but I just wanted to bring out some of the… may be if they were to improve, I don’t know it is not always possible to get a facilitated/positive journey in every field but I am glad that there was that element of linking even though that partnership or that advice, it didn’t walk through so it was one of my frustrating things in JAS1 this … -1:08:30 the conceptual framework but then you need to zone into your we objectives and they have to be workable and measurable. So yes you know you think you came with a concept but now you need to change it into a PhD but we need technical assistance at some point.

**Mod: She has shared with us the other part the negative part of it with the JAS, could you please share with us how CARTA has impacted on your PHD journey, this could of course be positive or negative. So you could still share with us on your research skills a number of you have already shared with us on your research skills, relationship with other researchers and your views about the research world, what have you got to say, how has CARTA impacted in those areas?**

R1: So I would still go back to where I started with my PhD journey, being known to be a CARTA fellow within the institution you get people just wanting to work with you, associate with you and you can begin doing things together. I can say when I began you know the fellowship, I didn’t have my skills but will just agree that now am at around such but with the research knowing how to write literally knowing how to do literature review not just review but critical review of literature. If you looked at my thesis at my masters level it was plagiarized let me just ….it is what I used, it is what I graduated from but seriously when I went through, when I ran through it I had like 50%... laughter

**Mod: this is after CARTA?**

P1: no, before

**Mod: you looked at it after ….**

R1: after I joined CARTA because at that point we were being taught how to critically review proposals, critically review literature it is through that you know the ground work of where you are starting your research from. I had a draft concept form but I had to turn it around literally from what it was to looking at things critically and I must say there was this whole six months journey of a cell.

If I can I mean I teach students proposal writing, literature review, I also teach research methods, I use a cell model of teaching in my teaching. Within the institutions we don’t have endnotes I teach through manual, I teach them how to cite, I teach them how to paraphrase no random copy and pasting because that is what I did, no one taught me how to do literature review and I can say within our institutions that is something that is really changing.

Okay my institution like I put it how can I do research and how can I do good research, the mentorship you get and I can’t say mentorship from you know the facilitators, I can say mentorship from each other, we learn from each other, somebody may not take you through the official mentorship process but working with someone knowing how they do things helps you work through the process, so it has been a good journey I can say so, a journey that has improved a lot in my life, both research wise and my teaching skills.

And I can say before they get to know you being a CARTA fellow, and by knowing this CARTA they can get to know your skill and for you also being a CARTA fellow gives you that, you know I know this now can I practice it, so you work with people, I have gotten to work with people to write grants of course you just get others rejected, but there are others that have been funded, I attribute all that to CARTA you know the learning process, but I can’t say; ‘’CARTA can just make you, you can make yourself through CARTA.’’

**Mod: thank you.**

P3: For Me we come from the same institution so the *receive* aspect of it is very critical and I learnt a lot, initially when I went back to the institution I was very curious about the plagiarism aspect so every time i would send a proposal I would request a soft copy then go through it, I run through it hand it in and I find everything is plagiarized so I became a bit critical and I at some point I felt like this is not fair to students and I am like probably what I am doing is not fair, so I requested that to be part of the team that teaches on ethics and plagiarism and all that because almost all the proposals that would come through my hand had issues, I would send it through and am like no, no, no, at some point and I would call the individual person discuss face to face basing on the same but then after that I would say probably I need to do something, so I just requested to schedule that let me be part of the team that guides students on that so we are working on that.

And one other aspect of recently I have learnt the peers, you know the colleagues as we are, the other day we were working on a project competing for something and I really learnt through my group members because we all divided work, am working on this another one is working on that and then after some time you meet together and critic, honestly it was enriching. Whether we win the thing or not at least I have the skill and my colleagues will bear witness with me at least they facilitated that.

P7: I just want to add more on what has been said and my focus will now be on the impact in relation to the collaborations, I think much of the collaboration has been covered in this JAS4 which I like but the missing part is about the practical aspect of it, and what I would wish is maybe deliberate efforts should be made where by the CARTA as a family avails the students with the help of the focal persons, create teams you know the partners they should be included, they should create teams and then help us to find I can’t say grants but may be opportunities which are there, which can attract grants so that we can go into the practical aspect of doing it. I know and I have said that we had an assignment to come up with a proposal to attract grant but I think this is not enough especially people like me whereby I have no knowledge and I know no knowledge on how to write even a grant. And what happens in most of the institutions is that when you are there you are not even incorporated what they expect is that you are going to look for yourself the grants and sometimes you don’t know even where to knock, so I think CARTA should strengthen with that. Yeah.

P7: Should I add something on that?

**Mod: yes it is okay.**

R7: because it is a critical issue especially when you have the focal persons in the various institutions, there are some focal persons from some institutions that are not active, I may say in institution it doesn’t seem to be active because at a point we seem to get lost like in the current cohort unfortunately we don’t have any one from my college which is very sad and I know there are many people who applied but the missing – you know at times when we have these focal people you get to know where you knock and all that.

And additionally you find that these focal people they have very active and huge clubs that are running but having time with the young upcoming researchers like us it is a big challenge so I don’t know if CARTA could influence whom to have as a focal persons in the various institutions because just picking any one because we have some who would never have any time for us, you look for them until you give up, at times they don’t respond to your emails, your phone calls, you know such collaborations would be very important for us and any upcoming young researchers …yeah

P5: just to support what she has said if it can be related to CARTA that they should work on the focal persons if possible they can change them they have done enough.

*[Laughter]*

P5: let’s have input from other people and at times if they try to get in another person they should try and make sure it is someone that can deliver because now the numbers of CARTA fellows in various institutions is on the increase and then that tells us that they have to be impactful and there is no way can make that impact if we don’t have a strong very active focal person. So if you have CARTA in a University as well and I have discussed with someone there that okay I think someone has done well so rest his case so some other person can also come.

[Laughter]

P5: so that is just to support what she has said and I am sure this will be a very good platform to relay our message to CARTA.

My impact, on the oversight I won’t say it is negative I would say it is challenging, it is because CARTA made me be on my toes, you know when you have fire on the mountain run run run, though the outcome is positive but then it makes you like my fellow participants have said that at times you are lost. There were times I wanted to face my supervisor and tell her that; ‘I feel that am tired, let me rest this PHD is not by force,’’ she has this mind of CARTA. I don’t know why she got to download the CARTA milestone and then she would be like; CARTA you have to do this, CARTA I said I need to work on the deliverables but can I have my peace? she followed me to the field, from another state to another state, ‘ I want to see your time line’ I want to see your timeline, after tongue lashing me she would pray for me, so when you are going to the field I want to come with you, you know that made me so crazy.

There was a time my husband came home and said you shouldn’t do anything in this house, your supervisor said you have to do this, wowawawa… I was like what is the meaning of this, this is a family affair PHD and CARTA there are three different entities from. Then my husband said no more roles here, I will do everything for you because your supervisor begged me that she doesn’t want you to be left out in CARTA. And I was like am even doing well, does he know?

And I think it is was because of - you know CARTA has these JAS deliverables that at times it makes so crazy sometimes you don’t put meals especially when it gets towards CARTA JASes and when you put the meal you will be like blar blar blar…

So think if they can….i don’t want to say they should take it softly but I think everybody doesn’t have the same coping mechanism so some of us have not even defended the thesis so it’s because at some point you just have to speak to yourself and say you know at this point take it easy .CARTA will come and CARTA will go so I don’t know may be if they can also work on the strategies so that they can bring out the best so I think for me it is was quite challenging.

Then JAS3, it was about data analysis and I had that time to work I think that was my best JAS, but then it was also so demanding, at the end of Friday you have to fill evaluation, I have to snap pictures so that I do not repeat something you know but all the best it is just to bring out the best in us which I think for the positive outcome.

**Mod: Okay thanks**

P6: For me actually just like any everybody has said it was challenging and I would say my first year in CARTA was crazy because I had a young infant to look after and sometimes through the night after working the whole day I had to work in the night trying to meet the assignments and all the rest at the same time I have to rest. But what kept me going was like; ‘’ you are doing work’’, actually personally it is challenging it has a lot of work but I work by the timelines because they keep me on my toes am actually doing a part time PHD because -53… a faculty member and there are so many things calling for or because I have deadlines to meet I actually had to sacrifice – I became a good time manager you know so many times I would be doing my things I would lock the door, some nights I would close the door just to meet the time line and I am sure if CARTA wasn’t there because the type of work I have now I wouldn’t have it because I had access to the best of the best globally. And even after the JASes I still have access to them so they have really been wonderful so in regards to the quality of my work, and for the training I have been able like I keep up with the guidelines.

And not only that I would say scientific writing, actually my skills have been sharpened, I can say these days the rate at which I get my publications out I have had the opportunity of publishing in some of the best journals out I think I have a higher … -52 and am sure it is because of those skills that I have been exposed to.

**Mod: maybe you could exhaust the JASes and everything because it has been brought out clearly, looking at the JASes that you had and they were around four, you had one in Nairobi, one in South Africa and I think one in Nigeria and this current one in Uganda, how could it be done differently? The JASes that you have talked about so let us look at the funding, the internship, facilitation, accommodation, program schedule and about that scientific writing and the payments that you had and the deliverables which the lady says was too much, so let us exhaust that so that we can always go back to this because it has come out clearly. Yes please?**

P6: Okay about the JASes, I think the gap between JAS two and three is a lot and actually the explanation they give is that they expect us to be in the field but at times it’s like we get lost, sometimes they just get some mails telling us we need this we need that. For example some deliverables were not clear cut, only for us to get mails like how many months to JAS3 telling us to have revised literature review which they never told us before so it was like some of us were taken a back and because of that there was some delay in submitting for that.

And if only I don’t know it may not be another JAS between JAS two and JAS three there should be like a major contact to us so that we will not get lost and lose count of time and more so better prepared for JAS3 so I think we need that major contact I don’t know how they are going to do it that but something major that will keep us on track and then we shouldn’t be getting mails without expecting specific such mails like those eseo (online scientific writing workshop) deliverable for JAS three, almost everybody was not aware, they were like taken aback, I was also taken aback that I actually had to suspend some things so that I would get it ready which I don’t think it was possible to get it ready.

**Mod: what do the rest have to share with us with the JAS, how can it be improved, what can be done differently to improve the different JAS that you have participated in, as it involves assignments you had I think assignments then you had also something to do with…is it.. for the JAS one you had I think the assignments isn’t it? JAS one and JAS two, they were three JAS. You had assignments and you also had to, I think JAS 3 is where you got the approvals to go the field for data collection,**

Chorus: it was…JAS3 it was just for analysis.

**Mod: just one**

P2: some people actually got their ethical clearance before JAS two and that’s another thing that should be clear because some of us had ethical approval before coming for JAS two and in JAS2 they were like trying to teach some things which wasn’t going to be possible. So I think it should be clear cut its either they want us to have ethical approval before coming to JAS2 or immediately after JAS two because I know a colleague who was actually told to change everything and he said at this point it’s very difficult I cannot change anything.

P5: Maybe we recommend that ethical approval should be taken after JAS two? Because during JAS two you have to develop the data analysis plan, you have to develop about analysis and how to go about the findings your methodology so I think it should be better off if we have it after, it is just my own recommendation such that people may know be – because by the time we finish JAS two we have now like almost over a year to come to JAS3 so I think that’s better. But I noticed during our – ethical approval was a pre requisite to JAS2.

*[All talk at the same time]*

P: the institution…

P1: this we appreciated but even when we go back to how JAS one starts people come in at different levels some come in one year to their PHD and PHD milestones so. Am wondering if someone who come in and has joined for two, three months, four months or even just about a year and they are in certain institutions which require them that after a year you must have done a certain…the CARTAs’ limitation then becomes an impediment to such a student who is going through the PHD within the school. They can improve other aspects but have already submitted their proposal and approved by the graduate committee it has been approved say by the ethics and now they are going through to do data analysis, then I don’t know, I know we get a lot and we learn a lot but we can change or even modify your proposal but I believe the modification can be used for amendments not but taking someone way back to re doing applications.

P4: I concur with that in fact I was under the impression in my institution for all you to qualify for CARTA you should have registered and I don’t know whether it is because .. it makes sense and I was under the impression that everybody is under the same umbrella but I was also shocked to find that by JAS one some people had presented their proposals and I pity them because most of JAS one now it is taking them back even gone back to the research question but you have already presented.

So but I don’t want to close doors for the future generations, i don’t want to say they should make a condition that don’t come for registration maybe it’s different institutions work differently but for our institution when you have registered for PHD you don’t qualify for CARTA and I think it is helpful and I think it avoids repetition.

And in the same grade for me JAS2 I was one of the thing where I felt like am stepping on one thing, JAS1 you are working on the objectives JAS2 again you know I felt like we didn’t move much I wish they could add something I honestly didn’t learn a lot, I was still doing the same steps as I was because by the end of JASone you are working on the objectives and methods now JAS two you are still on the same thing so I feel they could find a way of making it worth my work I thank God I was in my country but if I was in another country I would be like why did I come all the way if am still working on this so in my personal for me I feel for me I feel they could restructure to make it a next milestone that makes deliverables.

P7: just to add on what my colleagues have said about the JASes, I will talk about JAS two, JAS two the emphasis was on data analysis

P: JAS three

P: JAS two

P1: actually JAS two had research…

P7: we were introduced to stata, NVivo and made some practical but by that time as already elucidated by my colleagues others were just involved in their concepts, others involved in their proposals I think that was too late and we were even a month late to come out with the statistical data analysis which was difficult because you couldn’t visualize as one thing wasn’t done at that particular time. I know it is something to do with programming which involves a lot of partners but then if I was given a chance to propose maybe for JAS three instead of having four weeks it’s better to have maybe five weeks the other week to cover which was covered in JAS two about data analysis and then reduce the weeks in JAS two to three.

P5: Okay for me I feel JAS two it can be the revision of the curriculum because in JAS1 we were introduced to research methods and in JAS2 we were actually repeating and then I would prefer if we had more practical session on data analysis plan, different types of data analysis methods and which one will be suitable for your work so you know you have this skill, revising the objectives you have set, revising the methodology you plan to use, which of the data analysis plan would be suitable for your work? But then we had it like the tip of the ice berg at the time you had to - you just go for the one, you have them at the same time some of us had not made up our mind may be we would do purely qualitative or quantitative and when you say qualitative here, quantitative here so you don’t know which is which so I think they can if that is all about data analysis, methodology, research methods I think they should make it more of practical session I don’t have time for only one to sit down and give you reason why this then you try to understand the one that is suitable for your work.

And also well my colleagues from South Africa are here but I think I can also say that our facilitators at JAS two; should be quite friendly and also teach us like human, fine we were treated like graduate students, we are lobbying to graduate which is fine is okay but then the relationship you know addressing us it is a kind of whether you wonder what you have come to do. Some facilitators were very nice and they were approachable, some were treating us from a distance and so I think they can just work on them and see us as we are from different backgrounds and they should treat us as if we have different personality. But it is good that for the facilitators who were nice and we really appreciate them and also encourage those that are not too nice to just improve on their interpersonal relationship.

P6: for the data analysis plan as she has just mentioned I think it is very busy and they should have put more emphasis on it such that by the time we are in JAS2 we can now improve. So I think there should be a whole module on how to design a full data analysis plan and before we go for JAS2 we should at least have the first draft.

P3: so what I would like to add, I think my colleagues have mentioned a lot of things but I want to mention about JAS three, that during JAS three was it one or two where we had only experts on quantitative analysis.

P: week one

P7: so I would like to recommend that if possible would have both aspects those who are doing qualitative and quantitative at the same time because at times we switch at times you want to do quantitative you don’t get any time you decide to switch to qualitative but at that point there was no one although they made arrangements later but after a lot of time wasted.

Then the requirements to JAS three; as my colleague has said we shouldn’t be left alone for such a very long time then you are hurriedly told to submit this and this. And it would be good if they indicate all the requirements for JAS three and the time line so that we avoid cases of other cohorts being sent back because at times you find you reach there you do the diagnostics and realize that you don’t meet the requirements and they are sent back home that was not fair especially on the part of the fellows who had to go home. So we would like to recommend to CARTA they should simply indicate what is needed and confirm that before they give you all the air tickets and all that.

**Mod: I think we have shared more on the program, the way it was scheduled and how things were done under the JASe could you talk about the funding, internship, facilitators you have already talked about them I think those are the focal persons then the other facilitators when you come for the CARTA, is there anything you would like to add on that funding, internship, accommodation?**

P6: internship, I think it’s a good program and in my case I had the opportunity to attend the program in a particular area that is like new to me and had to work on and I was actually impressed by another partner who actually helped me to get that training so I think that was so good of CARTA because the knowledge that I got from there is actually what helped me in designing the particular part of the study I think that is a good thing.

**Mod: what have the rest have to share with us on that?**

P2: just to add on that the internships are really good maybe what I found was not enough was research money especially for Lab work it is not sufficient, yeah, may be if it is distributed somewhere else because you find that you are trying to get extra funding from elsewhere to meet your Lab requirements.

P5: me I benefited from attending a conference it was founded by CARTA so I think that is something I agree to and I also believe that so many of us though I didn’t go for the internship I know some people have attended internship so in terms of funding and the stipend always comes in due course and something that I always looked forward to. [Laughter] Now that we are about to get out from CARTA I am still asking myself that you know, no more stipends from CARTA.

And well it is a way of opening doors so I believe CARTA has helped me now I have to help myself and I think that for me I am so grateful for the funding, the stipends, the research funding, the conference funding it has really helped a great deal.

P4: I think we wouldn’t be fair to ourselves if we don’t comment about the accommodation for JAS three and they were limited to themselves with CARTA the fact they wanted to save money by building for CARTA, but it needs maintenance, the living arrangement was uncomfortable especially because actual teaching content is hectic you would at least want to sleep well, bathe well, to eat well not having to deal with cockroaches it is really important

Chorus: no lights, no water

P1: so it is yeah they really need to pull up their socks on that.

**Mod: was that in Nigeria?**

P4: in JAS three.

P5: why did you say Nigeria?

*[Laughter*]

P1: that is where JAS three was.

P6: I actually want to comment about that and I think CARTA can do better we all know that there is electricity problem in Nigeria so I think they can solve the problem by getting an inverter, you know, you have electricity in the room, and then having a borehole, I don’t think it is too much for CARTA, that’s how so many people survive, I mean you have water 24/7, I think CARTA should plan that in advance. I don’t think it is too much for them so that at least, we have sanity.

P5: just to emphasize that we started from safari park to no light, we put in on the platform, now it is better, I think, the feeding was superb we enjoyed the feeding…

P: probably CARTA wants you to experience all that.

[Different people are talking and laughing]

P1: maybe they should change the strategy they should start with that so that…

P6: the reason as to why I said CARTA could do better because even the poor don’t live under that so am wondering there is another place where you eat well, sleep well so if the poor can even live better then why CARTA, they can do better so that’s why am saying CARTA can do better.

P1: we must appreciate that JAS three was one of the best JASes we have done in terms of experience and output if that can be matched in terms of the accommodation for the JAS it would be one of the best JASes…

P7: probably somebody needs to do some research about JAS three because the conditions where we were, the circumstances, the food and running around with cockroaches as opposed to JAS two.

**Mod: you mentioned something that it was the best ever can you explain to us how it was the best ever?**

P1: I think it is one of the JASes where you can see the output, you go in with inputs and after coming out you either come out with the drafts; a draft manuscript, a draft of analysis, a draft of whatever, you would have done on analysis, it is a point where they leave you to find your way with facilitators around when you are trying to figure out where you want to go you have to reach out to.

And I think one of the JASes that is very favorable in terms of not so many lectures that comes along unlike JAS one, JAS two, JAS four where you have all the lectures. No one leaves you to go back to thinking about you about your research about what you want to do rather it’s been now these are the assignments you are going to do, so it was assignments, assignments, assignments unlike JAS three was literally few assignments and if it was assignment it was purely related to your own work something that makes you continue working on your own work.

P7: the other thing is about the funding the finances while we were in JAS three but I think the problem was communication, they could deposit your money and you are not told what the money is for. For example I was given money for the conference, even the amount they don’t send it unlike this time around, what I discovered I received half of my stipend so proper communication should be given, they can say that at this level when you are haven’t achieved the following deliverables you are going to receive half of your stipends. Because we kept on asking what is it? when are they going to give us?, … you ask is it for the thesis or what?

P: you can’t even plan for it.

P7: because some of us used the money for our research because the money was not enough so we need to know that. And the other thing is about publication they said that we need to publish in open access journal. Because for example the study that which am doing is in Malawi it is a different context from other countries and I want my people, to know more about that study rather than someone in Nigeria or South Africa and then they are saying we should publish in open access journal not your own journal because it is local so those things I think they also need to look at it at that particular country which is a reputable journal. And when we come to reading research issues people are very selective you can’t because you have published in a reputable journal the open access journal people are going to read it.

**Mod: Is there anything you would like to add, she shared with us the benefit of having been trained in scientific writing is there anything that you would want to be done differently so that it can be improved or you all appreciated what was done?**

Chorus: yeah we appreciate it.

P6: it has made us what we are.

**Mod: Okay let us go back to our previous question which we hadn’t exhaust before we ran into the JASes as it was causing a lot of… let’s again discuss, already you have mentioned some of these but could you please share with us how CARTA has impacted with your relationship with other researchers I have not heard that come out clearly, you know that there are a number of researchers other than Africans, other countries.**

P6: Actually in my institution now number one because which actually, number two they have seen my output I will say now people are now inviting me to join their research groups which actually was not - I didn’t have that experience before and not only that, I presented one of my work and I got an award for it and then few weeks after one of the focal person in CARTA contacted me that there was someone who wanted to work with me to actually write a proposal for one of the UK grants and it is because of my work. So I think that CARTA has opened doors, now I have been called upon to work in research groups not only that and even to collaborate at international level, so I think that’s a good cause.

**Mod: at least you have shared with us what happens in your institutions can you add more to that with other researchers what has been the impact of CARTA?**

P5: Okay for me in my institution now I collaborate with people from outside my discipline like now I have a research done working with people from demography and social statistics and that is quite far from my own department. And also now some time last year we wrote for a multi like a big a large population study to screen 10,000 women and that funding is under Bill and Melinda Gates and we got the funding. So now am working with other researchers from other disciplines we have O&G but I think am the only nurse, three of us are nurses and we work with the larger aspect of it the social workers and that has made me to CARTA really it’s like everybody wants to have a share of experience with CARTA, can you tell us about CARTA. Even this project that the grants we got they are going to write methodology aspect, we will be involved in the data analysis and that has really helped me.

And in the college some doctors will walk to you a lot, you can use NVivo, CARTA taught you how to use NVivo and this … eeehhh you know.

I have had opportunity to be invited to the research community of the university research center that they need my input and so I think CARTA just like everybody sees you like CARTA fellow, CARTA fellow. That also made me to want to put in my best because it is about people expecting you to deliver and you don’t want to cut down their expectations, so you make sure that you deliver, I think that is a good one.

P4: can I also share that for me it is actually this collaboration we got from JAS four i think this mentor mentee relationship even though they didn’t elaborate well but it is one of those surprises where we just got mails but I think it’s got a potential of creating collaboration by investing with other people so we know there is hope of a big collaboration.

**Mod: we are still talking about the impact that has really been part of the CARTA could you share with us about your views about leadership and understanding about mentoring, how has CARTA impacted on this?**

P1: leadership was brought up recently though we’ve been actors in our various institutions in various capacities we’ve led those teams, we’ve worked with people in terms of grant writing; I believe it is an aspect that is coming too late into our fellowship that now you have completed your PHD now you are becoming a leader. I think the aspect of leadership should also be chipped in as you go through your other JASes and not in the final JAS as a part of making you a leader, that is preparing me now to go and be a leader

P5: so now they are trying to say that now you are going, you are a leader.

**Mod: so it should have come right away.**

P5: you come out to say you know am a leader.

P6: it should be cutting across all the JASes.

Chorus: yes.

**Mod: I am not understanding about mentoring?**

P7: we have just seen it on the sketch mentoring of course we had it from where we are coming from but then.

**Mod: how has CARTA impacted on that?**

P7: okay I can’t say much because CARTA hasn’t introduced that to us of course we did it when we were doing other presentations issues came out but then it wasn’t like a topic to be, we haven’t learnt it yet.

**Mod: the next question I know you have already hinted on it also but could you share with us your CARTA journey about the low and high moments of CARTA?**

P5: the lows and high moments of CARTA, my high moments is when I received my stipends.

[Laughter, murmurs]

P7: they also brought some low moments because of the milestones and timelines, others had to catch up something like that others had responsibilities apart from being a student so to meet the two it was sometimes a problem.

R5: my low moment was eseo assignments, very many assignments, popping up of mails, so I now go back to my past and say you people have started again then having to meet all the deadlines, at times you open it you close it because you have other many things to attend to by the time it is like two days to the deadline, some people will request can you please give me some more time? you know those are my low moments but then coming to JASes these are my high moments when am at the airport going…yes.

P2: seeing countries.

P6: my high moment was when I got an award for my CARTA sponsored research. I really stood up, like, I didn’t expect it. And my low moments was the first two weeks of JAS two because I went with an infant and it was like there was no room for me within a week I changed accommodation two times, it was horrible that was my low moment.

**Mod: you can share with us your high moments and low moments**

P4: for me my institution expects me to continue with my daily job I lecture, I supervise my students honestly my high moments is actually this one month when I get to be away from my job and they are perfect I get to concentrate, and some of us when we want to concentrate you don’t want to be interrupted so I appreciate the fact that they managed to pay for us and take us away so that I go and do my things I really appreciate that is one of my high moments, of course leaving a family it is not always good but we have unlimited internet and technology, there is a way around it. So I think it was a good idea that they take us away to work and concentrate on your journey.

P2: for me one of my high moments was getting the CARTA award because when I saw the advert that circulated in the university that we could apply I discussed with my supervisor he said forget that one that one is Public Health people, am not in the core of public health people am in the other medical fields. So my supervisor discouraged me, that one? don’t even bother, it is for other public health people. So I just did it on my own, there were some tasks we had to do before we were selected and when I saw now we have reached this level we are waiting I don’t know for how long to know who the final people are, I didn’t have much expectation because my supervisor had already told me not to bother because that is not my field, so the day I got the email with the letter, I bought everybody lunch because we were walking to go for lunch with my colleagues the email came. So when I was trying I knew when I was not public health …so it was just a high moment.

The low moment was *eseo* there was a time when my daughter would be like mummy it is like you are not home because you are all the time busy, she leaves me sitting in the sitting room you are doing your work and you know at that point you even get irritable a child is disturbing and you really have a deadline, you have told Sebastian (*eseo facilitator*) to give you two more days … so yeah those were the bit of my low moments.

**Mod: those are what you have shared with us the low and high but can you share with us more on the entire journey of CARTA because you have had different some of what you shared with us were short lived let me say high moments but let us maybe look at the entire CARTA journey that you have attended several CARTA workshops should I say and other than that receiving the money that …what else, let us look at the whole procedure for example today you had a number of sessions what more can you share with us?**

P1: the whole CARTA journey or the PhD general journey, or the CARTA journey.

**Mod: CARTA journey I think has taken a bit of time, you have been to Nairobi, to South Africa, you have been in Nigeria, today you are in Uganda so it has been a journey and I think what you shared with us are those moments which have short lived and yet you have lived a journey a CARTA journey let me say.**

P1: I think that has made the journey itself because knowing that you have come from JAS one where you are excited I got this CARTA fellowship, and all that, then there is this carrot that they keep just putting in, there is stick and the carrot, there are ten assignments, those are really low moments. I remember I would just sit up also my baby is like I want to go to bed, then you are like okay let me put you to bed, you go the sleep just catches you, but you still have to complete the assignment so you wake up and your husband is there ooh I also want to go to bed, you haven’t done these assignments honestly that used to happen, it was a hustle so of course you get a stick sometime from xxx and the rest of the team. We worked through the process we have made it, we travelled we went to JAS one, JAS two, JAS two we have explained it was also another experience going back and forth not really understanding how different it is from what we did in JAS one viz a viz JAS two, and then you go in to this level, season no lunch…at least if the money comes but you are at limbo you don’t see the money

**Mod: you are at “limbo” meaning what?**

P1: no one, no contact, no one finds out where you are, it is literally almost a recess, they will only contact you on CARTA will only contact you when they need reports, give us a report and then no one gives you a feedback on those reports. Actually we need to know, you give a report but no one comes back to you to say ohhh by the way there are these things we think you are not doing well unless they get back to people who are not … Anyhow, so everyone is quiet, you are quiet then all of a sudden you wake up you are on your toes, you haven’t submitted this, then you are working eseo is there again, there then you are like…

[Laughter]

P1: and then you go to JAS three and get a shock of your life ohhh so this happens? We live through it we console each other we work through it and then the fact that both you are on PhD you are doing your PhD as well as CARTA expects you to publish you really need be on your toes, you really need to write, you really need to publish. So it is something that continues and I think everyone keeps saying we need you to publish we need to see your publications coming through.

And now when you come in to JAS four of course you are excited, it is the final JAS am not going for a month again but you are there thinking so what happens after this JAS and we leave each other, we may never get to see you know these countries but I think the climax process will probably make us work together, probably work in collaboration still visit each other in various countries. So that has been a journey for me and I don’t know if I share it with others.

P5: yeah, now that she has mentioned it, now I have low moment.

[Laughter]

P5: I am actually looking at her face and it is quite will I see them again you know it is always a time that always have time to interact learn so many languages, talk to each other, disagree and agree, so CARTA, CARTA has done well and like we said there is always room for improvement and which we have highlighted. But for me CARTA has really the journey so far has changed so far, has had some changes in my marital life, my spiritual life because at times I had to pray God help me with this assignment, God let me finish you know and even now that am finishing CARTA is like Lord what is the way forward, so spiritually. Then you know that it is also getting to me, CARTA has this you know writing grant, winning grant and you are like grant what do I write that will win grant so that makes me also pray those of us that believe in God that God help let me not fall from the hands of CARTA let them hear about me good news good news include me in those good news. And they give us motivations for the people that have won grants, this CARTA that I want so, so many million dollars, you are like where is this million dollars hanging how do I tap into it? So to me it is like a reflection on my spiritual life because at times you see these million grants as a miracle like some of us got the award like a miracle.

And also in my marital life you know I normally don’t live so long from the family I am a family person so coming to CARTA it has also changed my husbands’ perspective because he doesn’t want to leave his wife for so long because of domestic chores but then when I am not home I can see he will miss me, he will say I love you yet he rarely say I love you, good night I say good night,….take care of yourself, so for me maritally it has improved my relationship in my marriage we appreciate one another when I am really back from CARTA the first week you know I am like a queen, so beyond research, beyond academics my marriage, my spiritual life. And most importantly enter my busy plans…because when you have your money you can plan and move forward, you can plan for this you can plan for that, so even my children they all got to know CARTA, when I talk about CARTA they know CARTA they know what it is like, so every part of my family belongs to CARTA they join me together, they enjoy with me, like I said it is like a low moment for me that I will miss my people very soon.

Chorus: we shall also miss you too.

P6: I will personally describe CARTA journey as an onion, when you have an onion and you keep on peeling it layer by layer as it gets inside you get inside you have fresher leaves because each JAS I attend I keep on hearing about new opportunities, JAS1 and 2 I got to know so much about the internships training, JAS 2 they are also telling me about writing grants, because those are opportunities, it is a long designed program, and now I have told so many of my colleagues, I am like even if I go to do my PhD in outside countries I cannot have a better package because I have access to almost 100% of the facilitators, I write to them they will reply they will help me through not only that just relating with CARTA staff, sometimes you know opportunities can come your way for training or things like that, you get mails about conferences, about different sponsorship of opportunities, finding opportunities so I think this program if you have such opportunity in Africa then its good.

P1: if I can add something about the facilitators we come from institutions where professors are there you can’t reach them/not accessible, CARTA has brought its self, all its facilitators to our level you can call each other by first name, everybody will understand you every one will listen to you, you can work together, you can eat together on the table you can, you know in my institution when you go to the guest house, the mess there is a whole table for all the professors on the other side where they eat and then you have this others, where the commoners stay away, I have appreciated CARTA because of that where you learn…the professors that I meet here when i go back home they are just there, I believe…..[laughter] when you are here I call them by their first name.

P3: In fact there was a professor I was wishing he could come here, so that I could call him by his first name .. because I happened to have worked in ethical and research in the institution as an administrator and there was this document that we were looking at it we were assembling it so then he came to the office and I am like ooh we are sorry Dr.so and so your document is still under review then he starts “ whom are you calling Dr. so and so don’t you know I am a professor now”, then he sits and put the legs and says all that he thinks about me so the day I came here I was so scared, so I would first call everybody professor [Laughter] just to be on the safe side because I know missiles, then I got to know the names, you call others professor they say no, no, no, call me Helen, and am like ohhh so that’s what I really appreciate about CARTA.

P1: it is a humbling experience.

**Mod: you have shared a number of experiences with us I mean challenges that you have encountered with this fellowship, is there any other you could add rather than what you have discussed with us, is there any other challenge with this fellowship?**

P1: I think we have exhausted it.

**Mod: ladies can you share with us where you see yourselves in the next five (5) years?**

P1: grants, isn’t five too short?

**Mod: okay five to ten years (5-10) years**

P1: from five to ten years I would like to be a lead researcher or leading a grant let me just say that as a PI that is where I want to be.

**Mod: that is where you see yourself.**

P5: I agree with her.

P3: me too I want to be leading a grant and I would want to relink it back to CARTA when the opportunity comes because as they have mentored us through I would also want to see myself giving in.

P2: for me in addition to winning grants I see myself having a number of mentees because there are things which when I reflect how my Masters went in the kind of mentorship I got or probably not got I have learnt from CARTA what I would like to do in my institution that I have gained from here that wasn’t there. Yeah.

**Mod: ladies, where do you see yourselves in the next five or ten years to come that has gotten us far?**

P4: I am an academic and I know it involves research but the reason I wanted to get a PhD is because in our institution there is public health there was few and as a result I want to get a supervisor outside so I would like to improve capacity in my school as a mentor I don’t know whether it is mentoring or having mentees but yes strengthen that. And obviously I like telling my friends, my kids, my hope that one day I will be a professor, I have those aspirations, so am like publish publish , yes because am in this field I am hoping to do them. Of course doing grants it is nice it is signifying for me but honestly I want to be mentoring, just to increase our capacity within my school and department.

P7: I think it is the same issue with mentoring because coming with a background of being a nurse and then joining public health it was not easy as my colleague talked because in nursing even in my masters lead more to nursing and then some of the time I was blaming them for the first time especially something to do with the quantitative so my ambition is capacity building at my institution because most of the research which is done there is qualitative, people don’t want to do quantitative, quantitative for us no, so after going through the CARTA experience I hope I will be able to build capacity.

And again among nursing practitioners there is a gap a lot of them, they say it is something which is done by higher people who are doctors and professors, so I want to bring to their attention that research can be at any level and that can be achieved through capacity building, including the issue of publication, because when you talk of publication everybody says eeh, I know it is difficult they give critical comments so it is like a frightening experience.

P6: so me the next five to ten years, just like the others I see myself becoming a professor having research big grants, actually having research findings that can really influence policies not just in my country but Africa and even outside Africa, having many mentees that actually make an impact in the field because I have discovered that is a major thing that upsets my mind and that in the next ten years I see myself still remaining approachable, yes i wanna be free with my mentees, actually making a difference in the way Professors are seen.

**Mod: okay, is there any other thing you would like to add on that ladies?**

*Silence*

**Mod: okay thank you we have come to the end but if you have anything you would like to add on related to CARTA before we close or we come to the end I know you are rushing for the gym but…**

P5: we will miss CARTA fellowship, okay like she has said she would like to give back. I would love to give back to CARTA, coming for JASes if they have things where I can be consulted to add an input I will be willing to do that.

**Mod: thank you very much for your time ladies.**

P1: we would love to collaborate with all of you.

P5: lets’ wait for institutional grants.

**Mod: okay, thank you very much**.

**………………………………END…………………………….**
