# Supplementary material for: Experiences of doctoral students enrolled in a research fellowship program to support doctoral training in Africa (2014 to 2018): The Consortium for Advanced Research Training in Africa odyssey
Source: PLoS One. 2021 Jun 10;16(6):e0252863. doi: 10.1371/journal.pone.0252863 (PMC8191976; doi:10.1371/journal.pone.0252863)
Supplement: S3 Transcript — (DOCX) [file pone.0252863.s003.docx]

**CARTA FGD**

**DATE OF INTERVIEW:** 13^th^.03.2018

**CATEGORY OF FGD:** CARTA MIXED GROUP

**MODERATOR:** Robinah

**NOTE TAKER:** Resty

**TRANSCRIBER**: Resty

**NUMBER OF RESPONDENTS:** 07

**DURATION OF INTERVIEW:** 1hr: 30 minutes

**KEY**

**Mod:** Moderator

**NT**: Note taker

**P1, P2, P3**…: Participants

**Mod: our first driving question will be. Could you please share with us your experiences with CARTA? Each of you can share with us your experience before you could share the general experience.**

P9: For me I think overall we had an enriching experience with this consortium. We had our positives and negatives as expected but of course the gains we’ve registered cannot be taken for granted.

**Mod. Could you share with us more on the gains and the positives that you’ve talked about?**

P2: The training has been enriching definitely by the time we joined, the skills we have are really very obvious. We have improved in-terms of teaching; we’ve communicated in terms of beating timelines, all that has been a contribution of CARTA as I would say, the peers we always interact with from different background.

P5: I have appreciated the dynamic part of the cohort and also for ?the alternatives and also myself hadn’t I had that I might have fallen off the track but the peers dynamics have been a core part, the current family has definitely been forwarded my PHD and the resources all sorts of things happened and also costed those things of a single mum event but I don’t care with them taking on a PHD.

P4: I mean I can say that there have been more positives than negatives I don’t know what has been said previously, I think the timelines have been very helpful, otherwise things will not be done. We have to know that at such a time certain things have to be done. As always we interact with people from CARTA, you can actually get them back and speak to Justus and also from my CO Peter, to get feedback and it is also a two-way stream of communication not being a pattern of cohort but certainly a by-institution. The PHD road is very lonely, you often don’t have time to interact with other people especially if you are on full-time and being part of a cohort even if you teach as once a year, it gives you, you know being a whole-time professor Don and even other people’s successes gear you on as well.

**Mod: Others what do you want to share with us about your experience with CARTA?**

P2: I want to say that it has had good impacts, looking at the range of recipients that we have had to interact with in our cohorts have actually broaden my horizon, with respect to understanding how to relate with other people and how different disciplines can enrich research when you have different disciplines being involved in research project and that has actually helped me to appreciate that quite well.

P7: The CARTA fellowship and training has positively impacted my life, I have learnt and have developed skills on how to organize myself, beat deadlines, been able to work in a competitive environment with some other people to achieve set goals, I think that is a plus for me in CARTA.

**Mod: Is there anything that you would like to add on your experiences?**

P7: I have one more of the positives, it may not vary from the other positive outcomes. I think when we are in academic institutions, the goal was about getting a PhD, whereas in CARTA there are a lot of other key transferable skills like grant writing, networking and I think that introduced us to what we are doing now.

P6: One thing that I would like to add is that I really appreciate the visit to other countries on the continent, the fact that in different countries I had exposure to different sites and surveillance sites, different institutions and the context in which different countries do research and it has given me a lot more respect and understanding of how diverse the continent is, what the challenges really are and has made me much more experienced and has made me much more committed to not just the individual PhD but I would like to literally value people like my colleagues but it wasn’t really so before I started my PhD, so that shifted the kind of thinking that I had.

**Mod: Thank you very much for telling us, next we are going to start discussing the experiences we have had in particular and may be you could share with us your experiences since you are the last person.**

P8: May be as she is cooling down I can just bump in. My journey with CARTA has been largely enjoyable and I like the uniqueness of the program, diverse facilitators from all corners of the world who are leaders in the World. It has given me access to several colleagues from the African Continent. If I want someone in Rwanda, I can get someone, if I want someone in Nigeria I can get Bolu or someone else and you know it has taught us some practical issue, it’s not just to write a grant proposal, you actually have to go through the process, it’s not abstract you can actually go through the actual steps rather than being theoretical. And it’s not a program only just to produce PhD holders but actually to produce future scientist, so even my own supervisor, she has said, I am more confident, more decisive, I can argue I never used to do that before, I can’t say whatever the boss says is correct and they’ve really supported us. You know as researchers and one thing that I wish it would continue with us is to publish because it is very expensive to publish an article, it costs like 3000-4000 pounds which most of us don’t have even if I saved my salary, yet at least they pay for us.

Even if its outside my PhD it kind of brings us into it and the cohort issue, it induces a sense of benchmarking so, if my other colleagues have moved a certain stage, I also need to run back and its only here in such a situation that actually lies the capacity, at times you think, ‘’I cannot do this thing ‘’ but if your other colleagues are doing it, if they are winning grants, you can also win grants, if they are also publishing, you can also publish. The CARTA fellowship for me has been very supportive, be it IT (*internship*) and communication with people and they’ve done different cases, even if you are having personal issues, even if you are having challenges with your boss, then you can really take them to task, for some of the bosses maybe they don’t understand the agreements that they agree with CARTA because they agree that the fellows will have time but due to changes in their work place, they might forget about that but you know, CARTA has been really supportive for some of the fellows and I also like the seriousness with which they take these programs and they apply their rules very firmly and I’ve been in that position where I’ve had some challenges and I have seen more sanctions being implemented against me. But it’s not all against me but even across other fellows.

**Mod: Is there anything you would like to add?**

P8: Yes I would like to…my field is education, that I guess I have been selling that’s on capacity of everything and opened my mind in my horizon, maybe it has opened my eyes, mind but other areas, today we are super vibrant, you must be applicable. So, meeting people of different backgrounds is also teaching, so once the government emphasize on the teaching methodology as teachers, you know some of us are lecturers at the university, so we have learnt from the teaching methodologies, approaches from the facilitators we have come across in this journey, the JASes

**Mod: Can you add something?**

P1: I also think CARTA has improved the quality of my PhD, am not ashamed to say that I got my PhD from my Institution, because it’s not that my PhD is of poor quality because CARTA has allowed me to like go to different places, trainings, and some of the best schools in the World. So I think at the beginning choosing to study within in my country, it was a compromise, I thought it would be a compromise of quality with a lot of transfers but because of the circumstances with the home life, you can’t always leave to travel to study abroad. So CARTA was that compromise and I think it was a good compromise. Am not ashamed of the PhD, I know I have gained skills that other people haven’t gained even those people that have gone to the best schools because this thing about leadership, grants management, mapping stakeholders, I don’t think that is a typical for many doctoral studies. So, overall it’s been good.

**Mod: We have already shared what CARTA has impacted in the different areas of your journey to your PHD but there are specific areas where you would like to really pick more emphasis like your skills in research, how has CARTA impacted on it? Some of us have already hinted something into that area but could you throw more light on that? Research skills with regards to CARTA;**

P2: I think through the JASes there are key and tangible research skills like at the beginning just formulating what a question is. They even task us to know like ….. and use reference managers, but there were practical skills and even people who were not doing a degree and people did not know how to use Nvivo data management. So, there were practical things around that, how to define a research question. They even task us to know such managers and use references of the practical skills of even people who manages to do a hard degree but you just need to know how to use these handouts and people need to know how to use qualitative research and how to use NVivo because there were practical things around that, how to define the research questions and things like that and also getting contacts in forms of peers and facilitators that are involved in data analysis which will be online. Also one input as well is that I got to travel to an international conference where I got to present my work and my research pilot studies.

**Mod: The rest of us what do you have to add on your part as a result of CARTA, on your research skills and also the areas of relationship with other researchers not necessary from your institutions but outside other than your institutions?**

P3: To give an example, one other thing she has just talked about is the searching of literature, I think a lot of focus on this part of this systematic part of research whatever methodology is being used, but because one of the facilitators work with conflict for example have been able to be put together for example to assist the other managers part of a team and those kinds of opportunities to work on the people who are thorough and who could really help you. That kind of gesture is quite unique to our kind of opportunities but because of that there has been only one University to be able to give other PhDs and support team who are interested in putting together the results and talk through the processes and that even though I haven’t had that profession but I think those types of processes haven’t been useful in a sense that they have been reduced at least in my institution. In my University they encouraged the cities especially the first years on my university where they had to teach all courses applicable for instance I allow to take very seriously the teaching, I was not seen as an individual as from my faculty in our teaching research which would have allowed collaboration for many people who ask me to be a co-worker, I get disappointed with qualitative work. So, it’s definitely not something unnecessary i have to do with high efficiency and I didn’t know that I might do it when am a student but now it’s useless to us because I don’t have the confidence to do that.

P7: I think in the area of research I think CARTA has done a good job but it has won us some bit of research before joining definitely but it has empowered us to the extent that whenever you see an opportunity, you do not sit back. I can give an example, we have Havard coming to our University, coming to Makerere where they wanted to get people to work together. PIs (*principal investigators*) want people who are not very busy who are interested to work with them and they had established a network that was involving 7 Universities in Africa. So, I said okay, let me give it a trial but through this I’ve been able to work with them since 2015 to-date and we are given an opportunity to write small grants and then you win grants ranging from 5000 dollars to 10,000 dollars, you are given an opportunity to implement as a PI and you are really supported to publish your data. Before, coming up to say I am a PI was not really not very easy for me, I would go behind someone but now, to say I am the lead but now I cannot let such opportunities pass.

P1: What I would like to add, the research skills in my experience is slightly different because I feel like it was somewhat tilted towards qualitative researchers that even some of the methodologies and concepts were really around qualitative research whereas my PhD was completely quantitative despite the fact that you know CARTA was trying to encourage us to have a mixed method of study but mine wasn’t. So, I didn’t feel like I was getting as much just one out of two quantitative skills because the particular area that I wanted to focus on there wasn’t much people who could support. But realizing that, I found that at least I could go somewhere else, then CARTA facilitated me to go, so where they lacked some expertise, there is still an opportunity to have people who have the expertise. So finally they covered their deficiency.

**Mod: Is there anything you would like to add on that, on how CARTA has impacted on your research skills, the relationship with other researchers, your view on the research world, a few of us have shared with us that their teaching skills have improved, then also understanding about mentoring and about leadership. How has CARTA impacted on you in those areas?**

P4: for me I appreciate all partners of how CARTA has done the training and I gained research skills. I joined the CARTA Fellowship before I started my PhD and I joined it as an old school and I realized that they are offering diverse levels of skills. I had finished JAS 1 and I got more confused as I can put it, but I really appreciated what CARTA has contributed because even though I was confused and I could go back home and I rather sit down and I plan to see how I can probably settle together again. By the time I went for JAS 2 my skills in research had improved greatly. Even for literature reviews I had time to go over the literature review to see where I have gone wrong and to reconnect, back soon but …… had to refocus on my study, I had to change my topic twice, and to do that my supervisor said, ‘’yes I know you know what you are doing now” but I really had to discover the secrets, my skills in research improved greatly. I gained confidence to also mentor others. That was not part of me before.

To me my level of responsibilities is now in my head but I got to know later that I had this original responsibility to also impart on the students. So I got interested in colleagues and know how to tap opportunity and also students were our own army, so I was able to give extra compared to what I was giving before I joined JAS. These students at least they knew that some change has happened from somewhere else, but I saw such methodologies, and good ways of putting across messages that, ‘’no, it was not part of me before so I gained skills in those areas’’ So, it improved my research skills, it improved my teaching as a Lecturer. It also improved my ability to deliver, when I joined CARTA, I remember the same thing had happened to me when I joined my secondary school, I could dress my uniform and I made sure that I was dressed and when I joined CARTA I saw people from different countries and from different fields and I realized that not only people in Medicals are doing research, so I leant how to work with other people. In communication among the medicals, there is something I can gain, so we can work together, so I learnt that. So, when I got back home I was able to relate with people even outside my circles, make connection with people outside my circles and that improved me. And in the area of research there is an issue with people where I work, that also improved because I saw that in the behavior and attitudes of the international facilitators. But even when I see the people and experts and I feel that they are the high level of learners. You see the interest in partner, so I picked that from them also and those have been the good areas.

**Mod: Is there anything you would like to add?**

**ALL: silent**

**Mod: Could you share with us your view about the research world now that you are CARTA fellows? How do you view the research world, are you ready to join the research world? Are you most updated now that you’ve learnt through the CARTA program?**

P4: Yes if I can pick on from there the other time, I think I am more confident now to join the research world and as I’ve said earlier, I realized that you don’t do research by sitting in the corner of your room, focusing on your field which you would actually do before. Now in the research world you need to collaborate with people from different fields, people from different settings who will give you different context to your research. So you become leaders in your research rather than sitting down in a corner and doing your research and it is limited to you and your context. So, the capacity has been a big tool I think that is it for you to join research world. And I’ve also got knowledge and a bit of understanding that research is not limited to an area. So that is it.

**Mod: Yes please;**

P2: Am doing a PhD and CARTA has really contributed to taking me to the research world and I have changed to a large extent is not in line with junior PhD to research world and so that has been continuously surrounded by research world an interesting dynamic and it wasn’t with the stuff that I was doing on a daily basis.

P7: To me the concept of research world has changed through CARTA, I think I used to think of research just as a source of publication chains (smiled) whereas medicine researchers and I think CARTA have changed my thinking about research around. What it entails and the whole lot of policy making, I think composition and the roles researchers have throughout within society, in politics and having different communication outlets through the media, things like that. To me it makes me much more excited about it all, because I often come across researchers as more of a teacher or an activists and research was sort of underlined as a bad statement and to me it has become a much more essential place in my life and the cadres often make those linkages much more clearly and where I really feel like I will be centrally managing researchers at this stage.

So, I can go back to the leadership mentorship question. Well like I feel like CARTA has done a lot towards developing our skills and confidence around those things. I get more confused about the mentorship programs. For me IT wasn’t this stated clearly, mentorship relationship for this kind of programs, so I was never assigned to a mentor. Moreso, on the concept of things like this, I have my own ideas of the mentorship but the facilitators, different individuals excite me. They often say, ‘’I really like this, I like this style, I like this approach or I don’t like that or I don’t want to be that person’’ and I try to incorporate those things and it’s been less intentional and direct. For me, it is my experience of leadership. So to me mentorship has done the opposite and I feel very strongly where we’ve been asked to be mentors and get mentees and I really don’t know what I was supposed to do.

**Mod: Is there anything else that you would like to add about what she has shared with us about** **mentorship?**

ALL-silent

**Mod: CARTA has been a journey; we would request you to share what you consider as your low** **moments in CARTA journey and explain your high moments in CARTA journey?**

P1: JAS 2 was too rough, I think I don’t know whether it’s also a culture too, but it was true just as the South Africa, the way people interacted with us especially when they were giving us feedback on our studies, I think it would have been done in a kinder way, I left just too more confused than I was when I just came which I don’t think was necessary. And I feel like sometimes you know, not everybody is suited to this role, it doesn’t come naturally for everybody like most especially when you are like in a facility, especially when you are managing all the adults. So, perhaps there should be an orientation if there wasn’t, may be an improvement to how you deal with adults, especially keeping in mind in different context, even experiences over the years, we can step on each other just because you know like West Africans will take things at a certain way, East and Southern Africans are kind of close but you have to be like aware of the differences in culture.

JAS 2 South Africans just took it to another level. JAS 3 productively was the best but I felt that they could have done more in terms of accommodation. When I say done more I don’t mean a different venue but you know there are small things to me like cleanliness and like internet access and electricity. We really go through these challenges and they are already in our lives and I don’t think if you are to go through them just like there was no learning from that. So, I really didn’t mind the venue, it’s just that the preparation of the venue, you make sure the place is clean. If there are frequent power black outs, then have a generator because we came to work.

Then in JAS 4 as I said, I think we are dealing with people who don’t know how to manage adults but also if you want us to achieve a task, it loses meaning when it becomes something you just want to check over works, that ‘’ohh, they have submitted an abstract, check’’; they have done a policybrief, check; they have provided power points; check. No, I think we have passed that, it should have passed the quality of it and personally it’s at odds with what CARTA has been telling us throughout the years, telling us you know, it has to be quality work, it has to be, and then you come to a JAS that is over packed, giving you too many things, be a mentor, do that, we have 9 hours in class, after class you have to give an additional 3 hours to sort things out as you still have your own things. I think let CARTA come back to its principle which is quality and not about just checking things over a checklist that has been done.

**Mod: You said something about JAS 3 that was beneficial; could you elaborate more other than the** **accommodation kind of impact that was positive?**

P3: You find that the time to work on your research problem and then if you had a problem like looking for particular people to help you work on that particular problem, I guess to me the best thing about JAS 3 was the time. We had time which is lacking in especially the JAS 4

**Mod: I think let’s talk about the JAS, since she has emphasized on it, we can always go back to the other previous question because she shared with us I think, most of the low moments she has had, the fine moments she has had with CARTA as a journey. So, looking at the JASes that you have gone through, that is JAS1, JAS2 and JAS3, and I believe there were different activities in the different JASes that you received from different places, could you share with us, she mentioned something about accommodation, you could add on that, what exactly has been happening in the different JASes, then talk about the funding, internship, facilitator selection and may be program speaking?**

P2: I think what am going to say resonate with the previous question where I want us to share the high and low moments. What I have not really appreciated is the thinking and reasoning behind giving us the money for research. The amount of money we have been given for different researches I don’t think it is fair. I for one, I had to change my topic because I had to dance to the tune of the funder. At first I wanted to do something to do with adolescents in conflicting setting and all that, I realized that there is nobody willing to fund that. I quickly had to go into an area where everybody had an interest and was promised to support this global fund and this I did during my second year and also it was too drastic. So, I wish instead of saying that, ‘’one shoe will fit everybody’’ let them be considerate and review you know people with particular demands because even these young people, the first years, they keep on struggling with objectives to make sure that they get objectives that don’t demand a lot of fieldwork and that’s we emphasize also. In that way, they limit your innovation and you know, by the time you come to do PhD, some people already have interest in some areas but you find here that you are already in it. So, you have to switch from one discipline and almost to another discipline because of funding. So, I think this is an area we need to revisit critically (*banged the table*) If it cannot be done for the general population, then let them consider people with special interest to be assisted.

**Mod: You’ve shared with us the low moments that you have had with CARTA, their funding not fitting in** **what you really wanted to do, can you share with us your fine moments?**

P4: I have already shared that, the skills and everything.

**Mod: yes please**

P2: So JAS3 had a lot of difficulties that have been outlined, but the JAS was enjoyable but I think to me JAS3 is one other factor of a PhD candidate , somehow you are in the last of four years, and have a worldwide VHT. But at the end of JAS 3 you really have that sense of fulfillment and achievement that you actually finish, so that was a good feeling.

**Mod: For you what else do you want to share with us about JAS 1 and JAS 2, you have already shared with us how South Africa treated you, is there anything you would like to add on JAS1 and JAS 2 in relation to internship, facilitation, deliverables that you had to deliver, you had assignments in JAS 1 and JAS 2 and you had to come up with objectives (all laughed). And then you are comparing the different JASEs?**

P7: just to add on that one, JAS1 had a lot of repetition where you have to change the topics and all of that and I just think you just have to change and I would agree with other comments like my research was qualitative but as a student I had to distribute what was available and there is a lot emphasis on fitting the topics in the existing research because there is so much of repetition, given at a time that the biases that the grant was something like that but it had to be something viable and I think for some people as my colleague put it that it was their vision, what they will like in research for part of it to be moving forward, but there are strong biases in practicality.

Some things have been said as well about the funding had those kinds of things. And I think it was an unnecessary bias sometimes disappointinged them and I mean that we have friends and I think we do magnate and we have an idea that everything will have to change. JAS 2 is a bit over packed and in my experience and it is really important for things to move forward and we discussed it. It was all about imparting knowledge but I don’t know because there is need for feedback about South Africa experience. The over packed schedule, people have no time. They want to pack everything that you really have to master, a lot of that stuff, so that kind of reflection and you are supposed to a little bit of perseverance, you know that kind of thing. And then with others, JAS3 was my best, just a manger at the beginning like other beginnings had to see a light of it’s eternal pack. There is sort of dedicated space and time to work as individuals kind of and I was able to do what I needed to do. And I have the other colleague who says that JAS4 seems to be over ambitious, the truth is we need to have space.

**Mod: Gentlemen as you are so quiet what do you have to say in regard to JASES that you have gone** **through, what could have been done differently?**

P6: I would say that, maybe I would just put it differently; yes it’s true we got some heads up that we were going to be writing a grant in the first week but the situation is we have to submit our abstract, submit in our power point presentations, policy briefs and all that. We did not get the schedule for this JAS on time and so it was not easy for us to plan to have power point presentations before we arrived we’ve got feedback to be submitted in 5 pm persons before he arrived. So that by the time he gets we have time to focus on some of the tasks we were supposed to do while we are here. And I think some of us who go for JAS and am having one as well, I mean all the confusion we experience with some of these JASes would be avoided if we were given some form of advanced notice of what we are expected to do and submit a work plan while we are planning and we can plan better. I think that also goes to what someone said before. We need to be given a form of stand they know we can be given deadlines that we can always avoid a lot of pressure when they make us submit things which are not based on our abilities. That could be done better.

**Mod: Okay, is there anything that you can add on how something could be done differently regarding the JASes that you have gone through?**

P8: Thank you very much, I will start from JAS 4, yah it is very interesting. Some things that could be done differently, I think one thing is that I don’t know the lectures, I think we need more time, I know we have the quite a lot and I think we need a more dedicated place because there is also a lot to agree by the end of this. So, we too especially work and everything seems to be so packed with lectures and am not very happy, tying to do some of the assignments, I think that is very essential.

I also want to say something about food, I think the food should have some bit of flexibility for example assuming you just had a meal, the next food will come so early that you even can’t eat because it is just too close. Now when you get home, you will be hungry, right, and it is going to be a very long night, so that is also very essential. But JAS 3 is very interesting, we have a lot of space, we have a lot of support which I think is quite good. Now about, let me see if I can say move back and forth I can go back to something I said about in JAS4, I don’t know the JASes I will go to next but I think, but I don’t know why the others have been really organized and that’s why the schedule should be sent ahead of time, I don’t know if others have mentioned it and also the schedule was not there, so there was no prior preparation but from experience you just come in to discover some of the things. I think one last thing that I think is very interesting is that for me I can speak for myself, for something has been re-enforced. Now, another thing we are supposed to be running bout JAS4 again, I don’t know if I missed it, it is online. I think I didn’t get any reminders too.

**Mod: May be if I can ask you about the kind of support you received in JAS 3, what kind of support did** **you receive?**

P1: I think I have talked about the dedicated people, who move around, do you have any question? Unless like where we had somewhere to write. So that space to write is there to support us hence we’ve got support to help us in the process.

P4: I think that we got involved with a lot of JASes, JAS 1 I cannot remember, I just got overwhelmed because I was just getting into the system but I think JAS 2 and Jas4 could be different because of too much is not necessarily quality. Most of the people in this course are quite middle age but I would say they are old (all laughed), so their taking in squad is now very low, it would be very good if we could attend for 3 hours. When I was still an undergraduate I could wake up and then read the whole day throughout the night, now I cannot afford to do that.

So, these sessions already are over loaded, they should summarize and give us one hour to do whatever we think we can do. Of course there are points that some people are also aware of but they do not complain that it wasn’t given time. But most of us are working, that even though we are expected to do those major things like submit a Master’s dissertation for marking, I cannot ignore that I have to work. So if I was given that lee way, at least one hour in a day, you can share the day, but starting definitely at 8:00 and finish at 6:00 is not very helpful, we are tired during the day, during the night we cannot read. We are asleep (all laughed)

P4: and also people are active, they are always on social media

P2: So they should make it shorter and give us some free time to do X and Y and that’s why people like JAS3 because we were given a lot of some time like 3 hours just to work.

P7: I think the reason people are disappointed with JAS4 is that JAS4 is over ambitious, we have been here for just 2 weeks but people say JAS 4 is over ambitious. They want to put everything into your head (all laughed) but we know we work with adults and we are forced yet maybe what I can say is it is over ambitious(all laughed) and also a mistake that some of the facilitators are making, they assume that many of us have finished our PHD, the time we had JAS 3 we would have continued with it in JAS 4 so that they catch up with us, facilitating our PhDs out of our ability, so it should not be the other way round. So, for JAS4, they should continue with what they did in JAS 3, if you look at the notes in the 2 weeks in JAS3, a maximum of 2 or 3 facilitators at a time.

If you don’t have access then you could be accessed for anything, my work I read up to 3 in the night but I still work, to submit and finalize, I have to do it in the night and the night when am already tired and I feel sleepy. So, it is very difficult its over ambitious, JAS 3 to me is a highlight of the JASes where you have a time to work, where you would go home singing, yah when you are going home you’ll be singing that, ‘’ohh’’now am doing a PhD, JAS 4 when you go home you just need tea( all laughed) But in JAS 3 you say ‘’Ohh”, so we need to look at that and there are a few additions I would like to add on JAS 3 and JAS 1. Just as I have said I really never knew what I was doing in JAS1, I have forgotten what was done in JAS1, it was so confusing that I asked myself if I would leave doing this PhD. It was so confusing. So JAS 2 things could go better, JAS 3 the best and JAS4 I can say it was too busy, the first 2 weeks in JAS 4, I think it has not been a nice experience. So, they should revisit that in JAS 4.

P1: So, the other thing that needs to be put on board, Some courses I don’t think I call them Courses or JASeS, they need to shift to other JASeS, whereas of course if we are taking interest of policy, if part of it was taught in JAS 1 where the confusion they were talking about, it could help. So, when some parts of it have been taught in JAS1, the others in JAS4 because when they were preparing us to give in our recommendations. So another thing that I want to make is the facilitators on table. There are some facilitators in JAS 3, I don’t want to mention names, but the person who assisted me did not come in the first week. The first week I didn’t get assistance and I was equally slow, people should get assistance. So, I think they should have to look into their tables and they know all of this, am sure the fellows have talked all over.

P3: One thing in South Africa is that I could appreciate is that the MBH has a town where everything is on board, now it’s not quite often but I think sometimes these things come back. Some other things that we are sharing have already been said to CARTA. It is not fair ways where you don’t know what the people have to be. You don’t know what has been taken aftermath and one thing that facilitators look at. So we have to look at it as that but it rather is not appropriate but there are very good qualified with quality. My experience is okay to say that this person came individually and I appreciate the kind of leadership.

**Mod: Still going back to these JASes, can you throw more light on the confusion that was in JAS1, what was the confusion all about so that the next CARTA fellows that are coming in avoid those and in control of that program they avoid the confusion for those who are coming in?.**

P2: I think some of the confusion that was in JAS 1 is that most of the confusion is healthy but not like the one we had in JAS 4 if someone was asked he would say, there have been a lot of changes especially in the beginning of which I don’t think it’s confusion, I think it’s appropriate. If you came in to CARTA, you need to change things in the first 6 months that you start.

P7: May be partly why people think that people are confused or feel confused is that CARTA requires you to come with a concept with already supervisors sometimes assigned and before you join CARTA you’ve already agreed with your supervisor about what you are going to do. Here you come in and find experts, then they start telling you change that, change that and you find your supervisors from your institution tell you that if you change the topic, I will not supervise you. So, we have some bit of uncoordinated groups, I don’t know if they are changing, maybe they can have a teleconference with the supervisor, if at all the supervisor has been already selected.

**Mod: What can be done in order to address this confusion?**

P5: You know it’s quite different for those who didn’t have a supervisor at that time, then JAS1 was okay and I guess if you got to the people who had supervisors who were caught in that conflict, I think they should address it but for me JAS1 was okay.

P9: I will go to the part of the negative, I expect part of men, you are still sort of so big and assimilated, then some people come and just hold you on some of the negative but I think emerged because the things that I was trying to do that time and what I did was totally different. Just new things, all those new topics, the epidemiology, epistemology but I think those were just part of the courses.

P6: and I think that CARTA could have made sure that the people they are recruiting don’t get other topics and like they haven’t yet gotten themselves supervisors nor have registered a topic, because they want to have that influence in advance. People want to prolong I think they are now not accepting to prolong the disguise. It’s like I bring it from outside because you just go and make a goal and this probably is the whole program you see for you, others want to influence that, From JAS1 you have the supervisors but have to pay tuition. Someone says it has been the hardest thing that helps you to come to another institution. I think kind of trying to self-correct them and that causes the mistakes and the gray areas. I kind of agree that the confusion and that linking up is part of the process of their contract. These were too confident (laughter)

**Mod: Anything to add on the internship that you had during the JASes?**

P2: Okay after JAS3, I got an internship from CARTA to go for a training, so I had 6 weeks dedicated and it was very helpful, I also accessed my supervisors. It was a tough form of work because while at work it was sometimes difficult to combine writing with your lecturing. So, I think that was a very good initiative. And two, I leant many other people also access the protected writing thing as people call it. People should make sure that they maximize that period and then, they will come back with results that should be a kind of accountability for that period and for the course taken. So I was going to say, it is sort of experience.

**Mod: Is there anything you would like to add on the training you received on scientific writing?**

P6: I learnt so many new things about things like, open sentence, not letting your sentence go beyond the marks it was also a basic, I tried to teach the skills, so and I think that’s the goal of CARTA not just for you so that you can produce yourself and can in fact build capacity of other people. So, I think its support was greatly very welcome and I think it was a good experience.

**Mod: Is there anything you would like to add on the JASes with regards to payments of the different** **deliverables?**

P4: Well as to the deliverables, as it has been mentioned previously the fact that we had goals to attain has been very helpful, I am worried that now we are in JAS4 for those of us who haven’t finished and what will be the push you know to help us finish (all laughed) I mean yah, we are going to finish the course no matter what, it doesn’t feel that there is an alternative as long as you finish these 5 years, you are going to get a cycle.

P8: That’s why you finish, there are a number of things we have to do in terms of defending, and there are 2 units that we have to finish.

P7: and then when it comes to the money, for me I would say the money was okay, keeping in mind that ideally we are supposed to have another source of income, so I say it was very okay. It is less then what other fellowships provide but I think those other fellowships provide assuming that somebody doesn’t have any other source of income. And in case of me and my husband I just have to pay…. I don’t know the rest for the kids and building houses and obligation to stay as a family, I don’t know what the key source.

**Mod: Could you share with us I know you have already shared with us some of the challenges you have** **encountered during this fellowship, is there anything you would like to add on the challenges?**

P2: can I add something?

**Mod: Yes**

P2: I think like he said, the program is good but I think more attention need to be paid to fellows who are outside their country. Sometimes that extra fund that is to be paid does not come, you have to send e-mails, you have to follow-up, and you sometimes have to delay. If you come from Nigeria to South Africa to do research and you have to look for accommodation. But when that extra support does not come on time, of course it can put some pressure on you but that’s an area for improvement. I know it doesn’t affect many people but those who are affected I am sure they will appreciate what am taking about. So, that’s that.

**Mod: it’s okay. Looking again on the challenges you have encountered, is there anything you would like** **to add on that?**

P1: I will hesitate to say that it’s kind of one or 3people that it can be. So I had a baby during the JASes, between JAS 2 and JAS 3 and one thing which was quite disappointing is that though there is quite a lot of support with women with young children, I think one of the CARTA people was not comfortable that I had a baby and that was painful, so that was quite a very big experience in considering the fellowship because no one has spoken to me things like that especially that the patients havereproductive rights on their own. And it was not all that a big thing, it’s not like an important thing I have to report but that was just one of the things, but it may happen to one or 2 people (all laughed). This was from one of the most senior people and this was a very serious thing.

**Mod: Adding on what she has discussed, in the previous discussions that we had, it had also come out** **clearly most especially the ladies, should I say disturbances in the domestic women and having babies, is** **there any other experience may be you can share with us?**

P6: Yah I just want to talk about the policy to be able to bring up my child to JAS1 because it was such a relief. So, I appreciate it because that’s the only way I could have done the fellowship. So I really want to appreciate that opportunity, so that’s very responsible to bring to your attention, so that’s a positive and I had a bad family experience which affected my fellowship, my child and myself. It was so bad to the extent that I was really not sure I could continue and speaking to the leadership, they actually suggested to me a solution and I could take a leave. I one year to take care of my child and myself, and so it actually helped me as a single parent and brought my life back together. And again what was really important is that secondly I appreciated the kind of program, in that despite all that pressure, that I had and the kind of work that I do, I had the support I needed, though there are rules, I was treated as a special case, I had to follow it with all my life and I requested to be away and requested to come back for that program. But I appreciated that space to be able to do it at that moment, there were sort of more challenges like criticism. Whenever things that are personal happen in Africa especially when it affects family relationships, people have different perspectives. And I would like to say such criticism is not acceptable and often times I come to my classroom, somebody could say something unnecessary and that was very challenging for me particularly in the first JAS because I had to allow certain criticisms and see what happens to get supported. However certain cultures have been accepted for many years. So, that was something I had to face at some point in this fellowship program and that challenged me to be more accepting but there were some kind comments from fellows and facilitators. You know such criticism can affect someone else. Some comments were not so welcoming for me at that time. My husband and I had a problem. I recognize that people are from different background. The philosophy, is like CARTA has a philosophy and that not everybody not all the facilitators are tuned to that philosophy and I think, what other women have gone through those issues that I have just mentioned but also in the adult learning yet everybody needs to be on the same page because it does kind of sound experience when we miss people who Don’t, I guess you know what I mean by don’t.

P7: Dons form to the dons

P9: You know it kind of send messages to what CARTA is all about.

**Mod: Is there any other challenges that you have encountered during this CARTA journey? Gentlemen.**

P1: The only thing I want to add

**Mod: Yeah you can add**

P9: My fellow colleagues had mentioned it that you should have, I remember I had a grievance when I was in JAS1 (all laughed) and because of where am from, naming a baby is very important. Fortunately for me I still had some days to JAS 1 and I tried to make an official request and I learnt that you cannot be allowed to continue, so what I did was that I could go 2 weeks earlier and that enabled me to stay I CARTA. You start considering judgment, if you can’t afford judgment I had to call my parents to stand in for me and they said something that you can’t postponed it, so I went through a very good second year, there was flexibility because we are adults and people could ask me, ‘’did you go for the naming?’’ I did not go at all because previously I got information that, there was another fellow who also requested for 2 days and he was told to continue the next year because of children, I don’t think that’s fair enough for women, for men you have a lot of years that you can go for an MPH in CARTA, there are other issues about that, (all laughed) And that with the MPH I can say when I finish my MPH I will go back to my family.

P2: I disagree with the comment because CARTA is a fellowship, it has an achievement or what it needs to achieve. I think from your example you can’t compare yourself and others. They cannot take care of everything then we have to remember when we battle with this journey they told us that we have to make choices and there are certain things we have also to ignore. I agree I have a baby and a wife as well though am responsible for other things. The context of having a baby and nurturing the baby is not something done at school because (all laughed). I don’t think that you are supposed to have money to achieve this PhD, so now they are taking into play but someone suggests making a budget.

P8: If you are supposed to budget then that’s what, you combine, you had a baby while you are having JAS, You see, they are 2 different cases; you had a baby with how many Dons? (All laughed) So, I said a baby while having JAS so, requesting for a day or two for the father, ‘’ohh’’ they will not allow him because of his sons. But I don’t think 2 days out of one month I will not allow that or after 2 months, because someone may have gone home for a month. This is because CARTA cannot consider that we can accommodate that, we need data.

**Mod: the gentleman at the extreme end, (all laughed) what do you have to say may be about the** **challenges you encountered during this period?**

P1: Well I had some personal challenges, actually working around to combine my work schedule with CARTA schedule and the PhD. I have been a clinician and particularly a surgeon, it is quite demanding for me to be able to combine work. But I was personally worried about the CARTA program the as the main challenge. It was just kind of job that I do and still demand for another job. But I mean I tried to catch up with cooperating friends that I try to manage my time and my work better.

**Mod: CARTA fellows where do you see yourselves in the coming of the next 5-10 years?**

P4: Being a researcher, I will be able to earn my own money and be publishing and it will just be better (all laughed)

P5: Leaders in our respective institution I see myself as a change agent as facilitating the next generation of researchers and top class researchers and scientists from my institution. So I think CARTA has been providing platform to achieve that.

P2: 5-10 years, I see myself becoming a Don that’s the way I will put it. Certainly am able to become leader, a person that will be assisting the others a lot to get their career paths to see that the skills they have acquired are then taught.

**Mod: The rest of us where do we see ourselves in the next 10 years or 5 years?**

P5: As I said it is a journey, I also lead a team and I see myself and my collaborators winning funding and then I need a more disciplinary space, so I really target the challenges all over as I said African challenges with a stage to be led predominantly by the African side. Am not saying I will not be needing our partners in the US but we should also contribute more intellectual minds rather than you know bringing the funding. I don’t know what I should say but to contribute to the question of our problems in Africa and I agree that we need to change our institutions. We need to retrain others who have been trained, we need to train ourselves and also the academia is kind of a military institution but I think I need to contribute to say that a position is not taken because I come from Professor Chandiwana but being taken because of merit but not like coming from so and so as a highly graded professor, but actually does it make sense? Some of the things, they need to be challenged. There is no one answer, there can be many answers depending on how to address them.

P4: Well I see myself in my own position so that I can use the skills acquired using those JASes to up my research and also everything related to it.

P6: Just to add, I think all has been said but I also see myself as actually to being a research leader, doing research and influence policy is expensive and being a change agent in society, There has been a gap between research and societal impact. I look at the first 10 years to see research make impact in society and make such move.

**Mod: Yesterday something came out and I would like you to throw more light on the focal persons on your case finding impact, I want you to throw more light on them. In yesterday’s discussion there was a lot around the focal persons. Could you share with us your experience from your respective institution?**

P8: In my experience it’s been worse because we are called PI and I think I will focus then on sharing funds and so the focal person doesn’t play a very active role instead of engaging people and doing a role together and they understand their institution and fellows are registered in the school of public health a component where they might benefit a kind of program but I think the roles registered is that they are all coming from different institutions. Am not as other people but I make sure I have an active roles which people have, I don’t see much.

P9: Focusing on my school, I remember when I joined CARTA, it used to be hard work and links up but I think recently now we have to… I will talk about 2 different focal persons. You see why we had hard work, I didn’t consider by joining the pace. He was not somebody that I can say was approachable, highly qualified and lucky and slow pacing but initially I will prefer but we try when we are mature and with CARTA we are quite sufficient to involve colleagues to try to please go to him, that’s what I can say. Then when we adhere to, now I appreciate the person of the second focal persons. Now you are the one to decide whom you can decide whom to go to and who you can now work with. So, I believe the focal person should request their communication or silence them, may be increased frequency of their meetings but as you don’t have anywhere where you actually follow them after the CARTA program, you know they are making progress and ventilate their mind and tell them on time, before they issue the serious problem for them. I should not say it should be frequent. Still what they have to focus is communication and piloting with their CARTA program to make their progress and tell them on time. So they have issued a serious problem for them. So the meeting should be frequent in order to feel their impact. I think they should know why they work for communication, so if you are to see the focal person you are free, you see them.

P7: For me it’s difficult to say whether they’ve been more helpful oh not. We see them but I don’t think we talk with them. One, we don’t know their roles and we don’t know what kind of help we are expecting to get from the person. Here we have challenges in funding; you have to pay the supervisors you have to pay for entry. You don’t know what to share with the person. So, moving forward I think their roles need to be expressed, you know that will solve the problem. As to know whether they’ve been useful or not, it could be subjective. (All laughed)

P4: I would like to make a special mention to the support staff of CARTA. They help us study, they are the administrators, and I mean the people who are in charge in this country. They are really very accessible, they communicate with you, and I think they do a lot of work, we would like to say that, they have been really very amazing. You can access information, they show you the right direction of what you can do and where to get them, they are really good. So, even though they are other parts that don’t work there is one part that works and it’s across.

P3: I think this position is a bit informal. It’s drawn within their structure and I don’t know how much they are being remunerated. I think in this program, it could be made formal, so that apart from academic and all that; fellows need guidance on how to survive. So we need somebody to help them in order to survive, help them to do this, do that. That makes you comfortable when you have entered a foreign land. So, we need to formalize that position.

P2: I think being a student; we have different levels of persons

P3: Or Paul, we’ve been talking about Paul, these are guys who help us with the administrative survival

**Mod: Am emphasizing on some of these things since they appeared in the previous FGD so that we can also share idea on this. May be the last one, could you share with us about what you think about the timeline allocated to certain modules, sessions because we really want to state what really came out. So what is your opinion on the different time allocated to different modules? It came out clearly that some modules concerning analysis and packages are given more time than others, what do you have to say? Is it the time allocated to different modules appropriate or you felt they really needed more time than that?**

P4: It’s obvious to share about the JAS2 all where you have to go to packages, softwares, and it has to go back to those sessions but they were just too short. where you have to rule on 12, settle today before you understand what the exercise is all about daily and give tomorrow’ lessons, it is overwhelming. And practical sessions, even before you actually grab them and it’s so short, unless you have actually mastered the use of those softwares. So, it took me personally time after the JAS 2 to seek for some modules and during that time I was able to get my foot in using software. So, the time allocated, we should also look at courses that have practical aspect and it came outside to short. So the teaching and teaching hours of software courses of JAS 2 courses and one of adult scheme. Like so far we had a session, this was more of adult scheme. So JAS 2 had something to lay in the courses. If some sessions that are theoretical and abstract, they even make it very short. Teachings and teaching for hours doesn’t make sense (all laughed) something which will keep you alive, it’s not good to complicate issues. Even before setting this one you have already lost interest, that’s 23 hours, so it’s a two-way. You should look at the concept, the nature of the work, in terms of the African context. Some courses are so simple because you just have to emphasize what you have learnt, so they allocate you as per that course.

**Mod: Thank you very much. Do you have anything to add in regard to this discussion as we come to the end?**

P1: I just something to add, the cultural visit to South Africa was very good, and I hope you maintain that. But also you need to add something in Nigeria something that helps you to add context to your country. That would have also been good in addition to your country. I think it is something that adds to experience of CARTA, I don’t think we have missed such kind of thing.

**Mod: Okay thank you very much**

**……………………………………..END…………………………………..**
